# 3-acetyl-11-keto-*β*-boswellic acid and chitosan-Ag nanoparticles for synergistic tumor-resident bacteria mediated prostate cancer therapy

**DOI:** 10.1016/j.mtbio.2025.102374

**Published:** 2025-10-16

**Authors:** Bo Zou, Xuefei Tian, Ruisong Gao, Hongping Long, Yan Long, Bin Liu, Qing Zhou

**Affiliations:** a The First Hospital of Hunan University of Chinese Medicine, Changsha, 410007, Hunan, China; b Hunan University of Chinese Medicine, Changsha, 410208, Hunan, China; c College of Biology of Hunan University, Changsha, 410208, Hunan, China

**Keywords:** Prostate cancer, Tumor-resident bacteria, 3-acetyl-11-keto-*β*-boswellic acid, Chi-Ag NPs

## Abstract

Intratumoral bacteria play a critical role in prostate cancer (PCa) progression by altering the tumor microenvironment. Modulating these bacterial populations can significantly enhance the effectiveness of cancer therapies, including chemotherapy and immunotherapy. However, the use of antibiotics often yields inconsistent results due to poor targeting and the potential for bacterial resistance. In this study, we propose a novel therapeutic approach combining 3-acetyl-11-keto-*β*-boswellic acid (AKBA) with chitosan-coated silver nanoparticles (Chi-Ag NPs) to enhance PCa treatment efficacy by eliminating tumor-resident bacteria and inhibiting tumor invasion and metastasis. To address the challenge of drug targeting *in vivo*, we designed nanocomposites (SZTI01@Chi-Ag@PLGA@AKBA) that specifically target prostate-specific membrane antigen (PSMA) receptors on PCa cells. Compared to free drugs, SZTI01@APA NPs showed a 1.67-fold increase in accumulation at the tumor site. Once localized, the AKBA and Chi-Ag NP combination effectively inhibited tumor proliferation, induced apoptosis, and eliminated tumor-resident bacteria. Additionally, the nanocomposites suppressed Th17 cell infiltration and reduced IL-17 secretion, thereby inhibiting primary tumor growth and metastasis. In summary, this bacteria-targeting strategy enhances chemotherapy efficacy and immune responses, presenting a promising therapeutic approach for improving PCa treatment outcomes and advancing the development of more effective therapies.

## Graphical abstract

**Figure:**
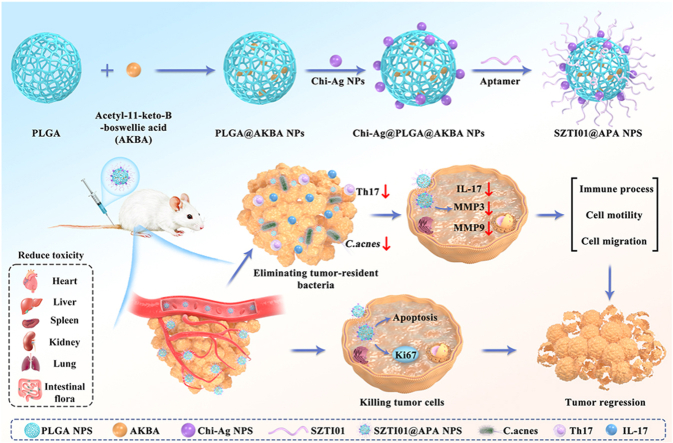

## 1 Introduction

Prostate cancer (PCa) ranks as the second most common cancer in men and is the fifth leading cause of cancer-related mortality worldwide [1]. Recent evidence suggests that intratumoral bacteria are an influential component of the tumor microenvironment, impacting cancer initiation, progression, treatment efficacy, invasion, and metastasis [2]. Microorganisms, such as those associated with bacterial prostatitis, can influence the onset and progression of PCa through both direct interactions at the tumor site and indirect modulation of immune responses [3]. Within PCa lesions, distinct bacterial communities have been observed to enhance pro-inflammatory responses, thereby promoting cancer development [4]. Despite these findings, microbiome-targeted therapies for PCa have not yet been integrated into clinical practice, underscoring the importance of further exploring tumor-resident bacteria as potential therapeutic targets.

The combination of chemotherapy and antimicrobial therapy offers a promising approach for inhibiting various tumors. For instance, Xi et al. demonstrated that antibacterial nanomedicines combined with gemcitabine could overcome drug resistance and suppress tumor growth [5]. Current antibacterial treatments, however, are primarily antibiotic-based, with long-term use leading to bacterial resistance and thus limiting effectiveness in cancer therapy [6,7]. Chitosan-coated silver nanoparticles (Chi-Ag NPs) present a promising alternative for antibacterial and antitumor applications due to their unique properties and safety profile [8]. In antibacterial treatments, Chi-Ag NPs have shown effectiveness in bacterial eradication and the prevention of bacterial resistance [9]. Moreover, Chi-Ag NPs can enhance chemotherapy efficacy by downregulating P-glycoprotein (P-gp) expression, creating a synergistic effect that inhibits tumor growth and metastasis [10]. Additionally, 3-acetyl-11-keto-β-boswellic acid (AKBA), an active compound from the traditional Chinese medicine frankincense, has demonstrated broad-spectrum antitumor efficacy and a favorable safety profile [11]. AKBA has been found to inhibit PCa cell proliferation and induce apoptosis [12]. These properties suggest that combining AKBA with Chi-Ag NPs could provide a powerful approach for targeting tumors and tumor-resident bacteria effectively.

Despite promising *in vitro* antitumor effects, the efficacy of certain drugs in clinical trials is often limited due to non-targeted accumulation, which reduces their therapeutic impact *in vivo* [13]. Recently, aptamer-targeted nanotechnology has emerged as a promising approach in drug delivery, enhancing therapeutic outcomes by specifically recognizing and delivering agents to target cells while minimizing damage to healthy tissues [14]. In this context, we constructed an aptamer-targeted delivery for AKBA and Chi-Ag nanoparticles using the emulsification-solvent evaporation and electrostatic adsorption methods. In this study, we utilized 2bRad-M sequencing to analyze tumor-resident microbiota in clinical PCa and normal prostate samples, revealing differences in microbiota richness between these groups. We then elucidated the mechanisms by which SZTI01@APA NPs exert therapeutic effects on PCa, providing a novel approach to combining chemotherapy and antimicrobial therapy in the context of tumor-resident microbiota in PCa.

## 2 Materials and methods

### 2.1 Materials

AKBA was purchased from Pufei De Biotech Co., Ltd. (China). AgNO_3_ was purchased National Pharmaceutical Group Corporation Chemical Reagent Co., Ltd (China). Chitosan was purchased from Sigma-Aldrich (MO, USA).Sodium borohydride (NaBH_4_) was purchased from Adamas-beta (China). The SZTI01 aptamer (5′-GCGTTTTCGCTTTTGCGTTTTGGGTCATCTGCTTACGATAGCAATGCT-3′) was synthesized by Accurate Biotechnology Co., Ltd. (China). Antibodies for rabbit IL-17, MMP9, and mouse MMP3 were acquired from Affinity (USA) Co., Ltd., while antibodies for Mo/Rt FOXP3, Mo CD25, mouse CD45, mouse CD4, and IL-17A were purchased from Proteintech (IL, USA).

### 2.2 Cell lines and animals

Human prostate stromal cells (WPMY-1), human PCa cells (22RV1 and DU145), mouse PCa cells (RM-1), human umbilical vein endothelial cells (HUVEC), vascular smooth muscle cells (VSM), human kidney cells (HK-2), and human normal liver cells (LO-2) were obtained from the Hunan Key Laboratory of Traditional Chinese Medicine Prescription and Syndromes Translational Medicine. Male nude and BALB/c mice were purchased from Silaike Experimental Animals Co., Ltd. (China). The animal experiments were conducted according to the ethical policies and procedures approved by the Medical Ethics Committee of Hunan University of Chinese Medicine (SYXK-2018-0006).

### 2.3 2bRAD-M sequencing of clinical samples

2bRAD-M sequencing was used to compare the relative abundance of microbiota in clinical PCa and normal prostate samples. This study was approved by the Ethics Committee of The First Hospital of Hunan University of Chinese Medicine (Approval No. HN-LL-GZR-2023-33), following the Helsinki Declaration and relevant local laws. Briefly, prostate samples were collected from patients undergoing biopsy at The First Hospital of Hunan University of Chinese Medicine. To prepare the 2bRAD-M library, species-specific 2bRAD-M markers were identified from a genomic database, with all sequenced 2bRAD tags mapped to the 2bRAD tag database using Perl scripts. The microbial taxa species across different samples were then quantified by calculating the total count ratios.

### 2.4 Effect of *C.acnes* on the invasion and metastasis of PCa cells *in vitro*

Wound healing assay: 22RV1 cells (1 × 10^5^ cells/well) were cultured in 6-well plate until reaching full confluency. A sterile 200 μL pipette tip was used to scratch the cell surface, followed by removal of the culture medium, washing with PBS three times, and incubation with culture medium containing different concentrations of *Cutibacterium acnes* (*C.acnes,* 10, 20, 30, and 40 MOI) for 48 h. Images were captured using a Nikon TI-S microscope (Nikon, Japan).

Transwell assay: 22RV1 cells (1 × 10^5^ cells/well) were cultured in 6-well plate and treated with C.acnes (10, 20, 30, and 40 MOI) for 48 h. The insert membrane (pore size: 8.0 μm) was coated with 20 μL of 10 % Matrigel. Next, 1 × 10^5^ cells in 100 μL of FBS-free RPMI-1640 were added to the upper chambers, while 700 μL of complete RPMI-1640 was added to the lower chamber. After 24 h, migrated or invaded cells were stained with 0.2 % crystal violet and counted.

### 2.5 Synthesis of Chi-Ag NPs

Chi-Ag NPs were synthesized using the protocol developed in a previous study [10]. Briefly, 500 μL of chitosan (10 mg/mL) and AgNO_3_ (10 mM) were each added to 8.5 mL of double-distilled water. After stirring at 1000 rpm for 10 min, 166 μL of NaBH_4_ (100 mM) was added dropwise to obtain Chi-Ag NPs.

### 2.6 *In vitro* synergistic effect of AKBA with Chi-Ag NPs

Cytotoxicity assay: 22RV1, DU145 and RM-1 cells were cultured in 96-well plates for 24 h. After the culture medium was removed, medium containing AKBA (10, 20, 30, 40 μM) or Chi-Ag nanoparticles (1, 2, 3 μg/mL) was added to 96-well plates, and the cells were further incubated for 48 h. Cytotoxicity was assessed using the MTT assay.

Association of AKBA with Chi-Ag NPs: 22RV1, DU145 and RM-1 cells were cultured in 96-well plates for 24 h. The medium was then replaced with 100 μL of medium containing AKBA (5, 10, 15, and 20 μM) and Chi-Ag NPs (0.75, 1, 1.25, and 1.5 μg/mL). The MTT assay was conducted to measure cytotoxicity.

### 2.7 Bacterial culture and agar diffusion test

*C.acnes*, a Gram-negative bacterium, was obtained from the Hunan Provincial Center for Disease Control and Prevention and cultured for 5–7 days at 37 °C in fresh LB medium. The antibacterial activity of AKBA (10 mM) and Chi-Ag NPs (10 μg/mL) against *C.acnes* was evaluated using the filter paper diffusion method. A 100 μL suspension of *C.acnes* (10^8^ CFU/mL) was spread onto agar plates, followed by placing a filter paper containing 25 μL of AKBA and Chi-Ag NPs. Control groups included normal saline, AKBA, Chi-Ag NPs, and AKBA + Chi-Ag NPs. Plates were incubated at 37 °C for 5 days, after which bacteriostatic ring diameters were measured. All tests were conducted in triplicate.

### 2.8 Antibacterial evaluation *in vitro*

The minimum inhibitory concentrations (MICs) of AKBA and Chi-Ag NPs were determined using the micro-dilution method. Briefly, *C.acnes* suspensions (10^5^–10^6^ CFU/mL) were incubated with various concentrations of AKBA or Chi-Ag NPs in a 96-well plate. After 5 days of incubation, bacterial viability was assessed by measuring absorbance at 600 nm. The MIC was defined as the lowest concentration with no visible bacterial growth.

### 2.9 Synthesis of (SZTI01@Chi-Ag@PLGA@AKBA NPs) SZTI01@APA NPs

Preparation of PLGA@AKBA NPs: The emulsion-solvent evaporation method was used to prepare PLGA@AKBA NPs. Poly(lactic-co-glycolic acid) (PLGA; 50:50, MW 24–38 kDa) was dissolved in DMSO (10 mg/mL) and then mixed with a solution of AKBA in DMSO (1 mg/mL). The mixture was sonicated at 20 W for 2 min and then added dropwise to 10 mL of a PVA solution (5 mg/mL). After an additional 10 min of sonication at 40 W, the emulsion was subjected to overnight dialysis (MWCO: 2.5 kDa) under continuous stirring, yielding a PLGA@AKBA NP (PA NP) suspension.

Preparation of Chi-Ag@PLGA@AKBA NPs: Chi-Ag NPs solution (54 μg/mL, 1 mL) was combined with the PLGA@AKBA NPs solution and sonicated at 20 W for 1 min. After standing at room temperature for 10 min, the mixture was centrifuged at 15000 rpm for 20 min, removing the supernatant and yielding purified Chi-Ag@PLGA@AKBA NPs (APA NPs).

Preparation of SZTI01@Chi-Ag@PLGA@AKBA NPs: For SZTI01 loading, the optimal drug-to-carrier ratio (1:9, w/w) was determined through adsorption studies. The Chi-Ag@PLGA@AKBA NPs were diluted to 900 μL with ultrapure water, followed by the addition of 100 μL SZTI01 solution (170 μg/mL). The mixture was incubated at 37 °C for 10 min under gentle agitation. Finally, the SZTI01-loaded NPs (SZTI01@APA NPs) were collected by centrifugation at 15000 rpm for 20 min at 4 °C, and the pellet was washed to remove unbound SZTI01.

### 2.10 Characterization of SZTI01@APA NPs

The diameter size and Zeta potential of Chi-Ag and SZTI01@APA NPs were assayed using Zetasizer Nano ZSP (Malvern Instruments). The Transmission Electron Microscope (TEM) images of SZTI01@APA NPs were observed by a JEM-2100 microscope (JEOL, Japan).

Elemental mapping was performed using SEM-EDS (Hitachi SU8010, 15 kV). Samples were prepared by depositing SZTI01@APA NPs on silicon wafers with 5 nm Au coating. Ag distribution was analyzed via Lα line (2.98 keV) using AZtecEnergy software, with acquisition parameters: 120 s/frame, 10 mm working distance.

The concentration of the SZTI01 aptamer within the NPs was determined using a 12 % polyacrylamide gel assay. Briefly, 10 μL of free SZTI01 and SZTI01@APA NPs (SZTI01 concentration: 2 mg/mL) were added separately to each well. The samples were run at a constant voltage of 100 V for 1 h, followed by silver staining to visualize the gel bands.

Drug release of SZTI01@APA NPs: SZTI01@APA NPs (75 μg AKBA and 50 μg Chi-Ag) was incubated in a dialysis bag (MWCO: 1000 Da) with PBS (pH 7.4) and immersed in 10 mL PBS solutions at pH 7.4 and 5.4. The setup was maintained in a 37 °C water bath. At predetermined intervals, 100 μL of the release medium was withdrawn and replaced with 100 μL of fresh PBS. AKBA release was quantified using Q-TOF mass spectrometry (Agilent, USA). Chi-Ag release was quantified using ICP-OES/MS (Agilent, USA).

### 2.11 Targeting and penetration of SZTI01@APA NPs *in vitro*

10 mg of Ce6 was dissolved in 1 mL DMSO and stirred for 6 h. Ce6 solution (1 mg/mL, 1 mL) was added to PLGA solution (10 mg/mL, 1 mL) and sonicated at 20 W for 2 min. The mixture was then added dropwise to a 10 mL PVA solution (5 mg/mL), followed by 10 min of sonication at 40 W and overnight stirring for dialysis to obtain PLGACe6 NPs. Chi-Ag@PLGA^Ce6^ NPs (AP^Ce6^ NPs) and SZTI01@Chi-Ag@PLGA^Ce6^ NPs (SZTI01@AP^Ce6^ NPs) were then prepared according to the procedure in section 2.9.

Cell uptake and targeting: 22RV1, WPMY-1, and PC3 cells (5 × 10^4^ cells per well) were seeded in 12-well plates and cultured for 24 h. The medium in each well was then replaced with 500 μL medium containing Ce6 or SZTI01@AP^Ce6^ NPs (final concentration of 10 μg/mL for Ce6) and incubated for 4 h. Cells were stained with DAPI for 10 min to label the nuclei and imaged using confocal laser scanning microscopy.

Deep penetration study: 22RV1 cell (5 × 10^3^ cells per well) was seeded in 96-well 3D culture plate (SPL, Korea) for 4 days. Ce6 and SZTI01@AP^Ce6^ NPs (10 μg/mL for Ce6) were incubated with 22RV1 spheroids for 24 h, followed by washing with PBS. Fluorescence within the spheroids was observed using confocal laser scanning microscopy.

Competitive Binding Assay of SZTI01 Aptamer: To validate the specificity of SZTI01-mediated cellular uptake, competitive inhibition experiments were performed. 22RV1 cells were seeded in 12-well plates (5 × 10^4^ cells/well) and pretreated with 17 μg/mL free SZTI01 aptamer for 2 h at 37 °C to saturate binding sites. Subsequently, SZTI01@APA-Ce6 NPs were added and incubated for 4 h. Cells were stained with DAPI for 10 min to label the nuclei and imaged using confocal laser scanning microscopy.

### 2.12 Targeting and penetration of SZTI01@APA NPs *in vivo*

Tumor targeting and penetration assay: Inoculate 1 × 10^7^ 22RV1 cells (100 μL per mouse) subcutaneously into the left axilla of 4-week-old male BALB/c nude mice to establish a subcutaneous PCa xenograft model. Inject 200 μL of culture medium containing free Ce6 and SZTI01@APA NPs (Ce6 dose: 2.5 mg/kg) via the tail vein. Use the IVIS® Lumina system to acquire Ce6 fluorescence images at 6, 12, and 24 h. Mice were then sacrificed, collect major organs (heart, liver, spleen, lung, kidney, and tumor), and perform fluorescence images to analyze the biodistribution of Ce6 in different tissues.

Pharmacokinetic assay: Inject 200 μL of Ce6 and SZTI01@APA NPs (Ce6 dose: 2.5 mg/kg) via the tail vein into 8-week-old male BALB/c mice. Collect blood samples (100 μL) at 0.5, 1, 2, 3, 4, 6, 8, 12, and 24 h, centrifuge at 12000 rpm for 3 min to obtain the supernatant plasma. Fluorescence images of the plasma were captured using the IVIS® Lumina system.

### 2.13 *In vitro* anti-cancer activity of SZTI01@APA NPs

Cell viability: Cells were seeded in 96-well plates (5 × 10^3^ cells/well) and cultured for 24 h. Then, cells in each well were treated with AKBA, Chi-Ag NPs, AKBA + Chi-Ag NPs, or SZTI01@APA NPs, all at equivalent concentrations of AKBA (15 μM) and Chi-Ag NPs (1 μg/mL). After 48 h, cell viability was assessed using the MTT assay.

Apoptosis assay: 22RV1 cells were seeded in 6-well plates (1 × 10^5^ cells/well) and incubated at 37 °C with 5 % CO_2_ for 24 h. Cells were then treated with AKBA, Chi-Ag NPs, AKBA + Chi-Ag NPs, or SZTI01@APA NPs at the same AKBA and Chi-Ag NPs concentrations (15 μM and 1 μg/mL). After 24 h, cells were stained using the Annexin V-FITC/PI Apoptosis Detection Kit (Tianjin Sungene Biotech Co., Ltd., Tianjin, China) and analyzed by flow cytometry.

3D tumor sphere model assay: 22RV1 spheroids were treated with AKBA, Chi-Ag NPs, AKBA + Chi-Ag NPs, or SZTI01@APA NPs at the same AKBA and Chi-Ag NPs concentrations (15 μM and 1 μg/mL). Images were captured on days 1, 3, 5, and 7 using an inverted phase-contrast microscope.

### 2.14 Assay of cell proliferation, migration, and invasion

Wound healing assay: 22RV1 cells were seeded in 12-well plate and culture until the cells reach confluence. Using a sterile 200 μL pipette tip, create a scratch on the cell surface. Remove the culture medium and wash the cells three times with PBS. Then, incubate the cells with medium containing PBS, AKBA, Chi-Ag, AKBA + Chi-Ag, and SZTI01@APA NPs (AKBA and Chi-Ag concentrations of 15 μM and 1 μg/mL, respectively) for 48 h. Finally, take and record images using an inverted phase-contrast microscope (TI-S, Nikon, Japan).

Transwell assay: 22RV1 cells (1 × 10^5^ cells/well) were seeded in 6-well plates and treated with AKBA, Chi-Ag NPs, AKBA + Chi-Ag NPs, or SZTI01@APA NPs at the same concentrations (15 μM and 1 μg/mL). After 48 h, 100 μL of FBS-free RPMI-1640 (5 × 10^4^ cells/well) were added to the upper chamber of 24-well inserts (pore size: 8.0 μm) for migration analysis. For invasion analysis, the insert membrane was pre-coated with 30 μL of 10 % Matrigel (Corning Matrigel Matrix, US). 22RV1 cells (1 × 10^5^ in 100 μL FBS-free RPMI-1640) were added to the upper chamber, with 700 μL of complete RPMI-1640 in the lower chamber. After 24 h, migrated or invaded cells were stained with 0.2 % crystal violet and counted.

### 2.15 *In vitro* antibacterial evaluation of SZTI01@APA NPs

PBS, AKBA, Chi-Ag NPs, AKBA + Chi-Ag NPs, and SZTI01@APA NPs (with AKBA and Chi-Ag NPs at concentrations of 15 μM and 1 μg/mL, respectively) were incubated with C.acnes suspension (10^9^ CFU/mL) at 37 °C for 12 h in a constant temperature incubator. The mixture was then centrifuged at 8000 rpm for 5 min at 4 °C to remove the supernatant, retaining the bacterial-material precipitate. The precipitate was fixed in 2.5 % glutaraldehyde for 4 h in the dark, and then dehydrated through an ethanol gradient (30 %, 50 %, 70 %, 85 %, 95 %, and 100 %). Bacterial cell membrane morphology was observed using a scanning electron microscope.

### 2.16 *In vivo* anti-tumor effect of SZTI01@APA NPs

22RV1 cells (5 × 10^6^ cells in 100 μL PBS) were injected subcutaneously into the right flank of 8-week-old male nude BALB/c mice. Once tumors reached approximately 100 mm^3^, mice were randomly assigned to five groups (n = 5): (a) PBS, (b) AKBA, (c) Chi-Ag NPs, (d) AKBA + Chi-Ag NPs, and (e) SZTI01@APA NPs. The administered doses of Chi-Ag NPs and AKBA were 0.75 mg/kg and 5 mg/kg, respectively. Inject every other day for a total of 7 doses, lasting a cumulative period of 12 days. Tumor volume (V = length × width^2^/2) and body weight were recorded every 2 days. Tumor inhibition rate (TIR) was calculated as TIR = (VPBS - V_t_)/VPBS, where VPBS represents the average tumor volume in the PBS group, and V_t_ represents the average volume in other groups. At day 12, tumors were excised, fixed in 4 % paraformaldehyde, and embedded in paraffin for H&E staining, TUNEL staining, and Ki67 (1:5000) labeling.

### 2.17 *In vivo* anti-*C.acnes* and anti-cancer effects of SZTI01@APA NPs

To evaluate the dual anti-*C.acnes* and anti-cancer effects of SZTI01@APA NPs, a PCa axillary RM-1/*C.acnes* tumor model was established. RM-1 cells (5 × 10^6^ in 100 μL PBS) were injected subcutaneously into the right flank of 8-week-old male C57BL/6 mice, with *C.acnes* (100 μL, 10^7^ CFU/mL) injected around the tumor at multiple points every 3 days (3 injections total). Mice were divided into three groups (n = 8): (a) PBS, (b) *C.acnes*, and (c) *C.acnes* + SZTI01@APA NPs, with Chi-Ag NPs and AKBA doses at 0.75 mg/kg and 5 mg/kg, respectively. On day 12, tumors were harvested for flow cytometry analysis. Tumor tissues were homogenized to 1 g/mL, diluted 10-fold, plated on agar, and incubated anaerobically at 37 °C for 7 days. Colony counts were observed, photographed, and analyzed quantitatively using Image J.

### 2.18 Transcriptomics

To investigate the mechanism of SZTI01@APA NPs in RM-1/*C.acnes* tumor model, tumors were collected for RNA sequencing. Total RNA was extracted (TRIzol), and poly(A)-enriched libraries were sequenced (Illumina NovaSeq, 150 bp paired-end). Bioinformatics analysis included alignment (HISAT2), differential gene expression (DESeq2, |log2FC|>1, FDR<0.05), and pathway enrichment (KEGG, GSEA). Statistical significance was determined using one-way ANOVA (p < 0.05).

### 2.19 16s rRNA sequencing

To investigate the impact of SZTI01@APA NPs on gut microbiota stability in the RM-1/C.acnes tumor model, fecal samples were collected from mice at the conclusion of the experiment. Microbial DNA was extracted (QIAamp PowerFecal Kit), and the V3–V4 region of 16S rRNA was amplified (341F/806R primers) and sequenced (Illumina MiSeq). Data were processed (DADA2, QIIME2) to assess α/β-diversity (Shannon, PCoA) and taxonomic shifts (LEfSe).

### 2.20 Toxicity of SZTI01@APA NPs *in vivo*

Male nude BALB/c mice were randomly divided into five groups (n = 3): (a) PBS, (b) AKBA, (c) Chi-Ag NPs, (d) AKBA + Chi-Ag NPs, and (e) SZTI01@APA NPs. The doses of Chi-Ag NPs and AKBA were 0.75 mg/kg and 5 mg/kg, respectively. The injections were given once every other day for 7 times, and the cumulative duration was 12 days. After 12 days, blood samples were collected from the nude mice for routine blood test and liver and kidney function test. After euthanizing the nude mice, the main organs (heart, liver, spleen, lung, and kidney) were taken for histopathological observation by H&E staining.

### 2.21 Zebrafish toxicity assessment

Zebrafish embryos were exposed to SZTI01@APA NPs (0, 50, 100, 150, and 200 μg/mL) in E3 medium (n = 10/group) under standard conditions (28.5 °C, 14:10 light/dark cycle). Morphological changes were recorded at 0, 24, 48, and 96 h using bright-field microscopy. Survival rate was determined by counting viable embryos with cardiac activity at each timepoint. Heart rate (ventricular contractions/min) was measured in 48 h embryos, while body length (rostral-caudal distance) was quantified at 96 h using ImageJ.

### 2.22 Statistical analysis

Details of statistical tests, *P*-values, and the number of replicates are provided in the figures. A significance threshold of *P* < 0.05 was applied for all experiments. Statistical analyses were performed using GraphPad Prism 9 software.

## 3 Results and discussion

### 3.1 Highly enriched *C.acnes* in PCa tissues

**Fig. 1:**
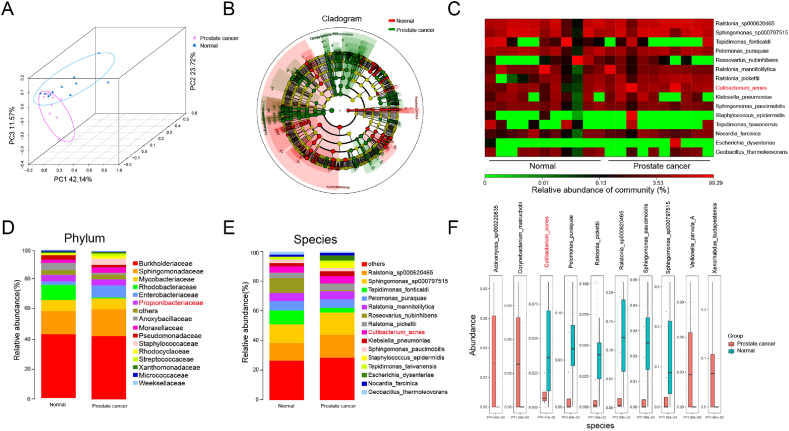
**Differential abundance of resident bacteria between PCa and normal samples. (A)** Principal Coordinate Analysis (PCoA) reveals differential tumor-resident microbiota structure composition between the PCa group and the Normal group. **(B)** Differential species annotation branch diagrams, with green nodes representing species with significantly higher abundance in the PCa group and red nodes indicating higher abundance in the normal group. **(C)** Heatmap illustrating species-level differences between PCa and normal groups. **(D**–**E)** Boxplots illustrating the differential abundance at the phylum and species levels between the PCa group and the Normal group. **(F)** Boxplot of the top 10 deferentially abundant species. (For interpretation of the references to colour in this figure legend, the reader is referred to the Web version of this article.)

Risk factors for PCa include age, race, and family history [15,16]. Recent studies suggest that certain microorganisms may induce DNA damage, contributing to tumor formation and progression [17]. To investigate the types of resident bacteria in PCa tissues, we collected 10 PCa and 11 normal prostate samples post-biopsy and analyzed them using 2b-Rad sequencing technology. Principal Coordinate Analysis (PCoA) revealed significant separation between the bacterial communities in cancerous versus normal tissues, indicating distinct differences in bacterial abundance between PCa and normal prostate tissues (Fig. 1A). Annotated branch diagrams further illustrated that the proportion of green nodes (PCa tissues) significantly increased compared to red nodes (normal prostate tissues), highlighting an overall increase in species abundance in PCa samples (Fig. 1B). Heat maps and bar charts based on bacterial abundance at the family and species levels showed a marked increase in the *Propionibacteriaceae* family and *Propionibacterium acnes* in tumor tissues (Fig. 1C–E). Box plots of the top 10 most abundant microbes, excluding hospital-acquired infection bacteria, confirmed a significant increase in *C.acnes* levels in cancer tissue (Fig. 1F). Consistent with previous findings, *C.acnes* was more frequently detected in PCa patients than in controls (60 % vs. 26 %) [18]. Additionally, several studies report elevated levels of regulatory T cells (Tregs) in PCa tissues containing *C.acnes*, suggesting that *C.acnes* may promote an immunosuppressive environment conducive to PCa onset and progression [19,20]. These findings underscore *C.acnes* as a potential therapeutic target in PCa treatment [21].

### 3.2 *C.acnes* promotes invasion and metastasis of PCa cells

**Fig. 2:**
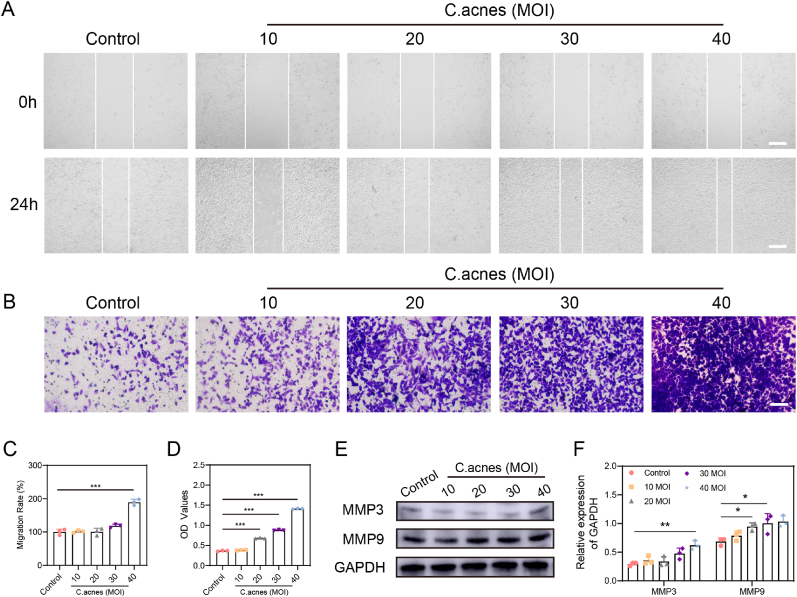
***C.acnes*-induced expression of MMP3 and MMP9 promotes invasion and metastasis of 22RV1 cells. (A)** Images of scratch wounds in cells treated with PBS and *C.acnes* for 24 h in 22RV1 cells. Scale bar: 100 μm. **(B)** Number of invaded cells using transwell chambers after treatment with PBS and *C.acnes* for 24 h in 22RV1 cells. Scale bar: 100 μm. **(C)** The wound healing rate of 22RV1 cells treated with PBS or *C.acnes* for 24 h. **(D)** Absorbance values at 590 nm after dissolution of crystal from transwell chambers in different groups treated with PBS and *C.acnes* for 24 h in 22RV1 cells. **(E**–**F)** Western blot analysis and Image J quantitative analysis of the effects of *C.acnes* on the levels of MMP3 and MMP9 in 22RV1 cells after 24 h.

The tumor-resident bacterium *C.acnes* may contribute to cancer progression through the production of active metabolites [22]. We conducted wound healing, transwell, and western blot assays to evaluate the effect of *C.acnes* co-culture on the invasiveness and metastatic potential of 22RV1 PCa cells. In Fig. 2A, a high concentration of *C.acnes* (40 MOI) significantly enhanced the wound healing rate of 22RV1 cells. Compared to PBS, wound healing rates increased by 1.7 %, 0.5 %, 18.6 %, and 89.3 % at *C.acnes* concentrations of 10, 20, 30, and 40 MOI, respectively (Fig. 2A and C). The number of invading cells in the transwell assay also rose with increasing *C.acnes* concentrations from 20 to 40 MOI (Fig. 2B and D). Matrix metalloproteinases (MMPs) are zinc-dependent enzymes that degrade extracellular matrix components, facilitating tumor cell invasion and metastasis [23]. Western blot analysis revealed that *C.acnes* at 40 MOI increased MMP3 levels by 1.5-fold, while MMP9 levels rose by 26 %, 32 %, and 33 % at 20, 30, and 40 MOI, respectively (Fig. 2E–F). These results indicate that *C.acnes* may enhance tumor cell invasion and metastasis in PCa by upregulating MMP3 and MMP9. Thus, targeting tumor-associated *C.acnes* could be an effective strategy to reduce PCa invasion and metastasis.

### 3.3 AKBA synergizes with Chi-Ag NPs to kill PCa cells and *C.acnes in vitro*

**Fig. 3:**
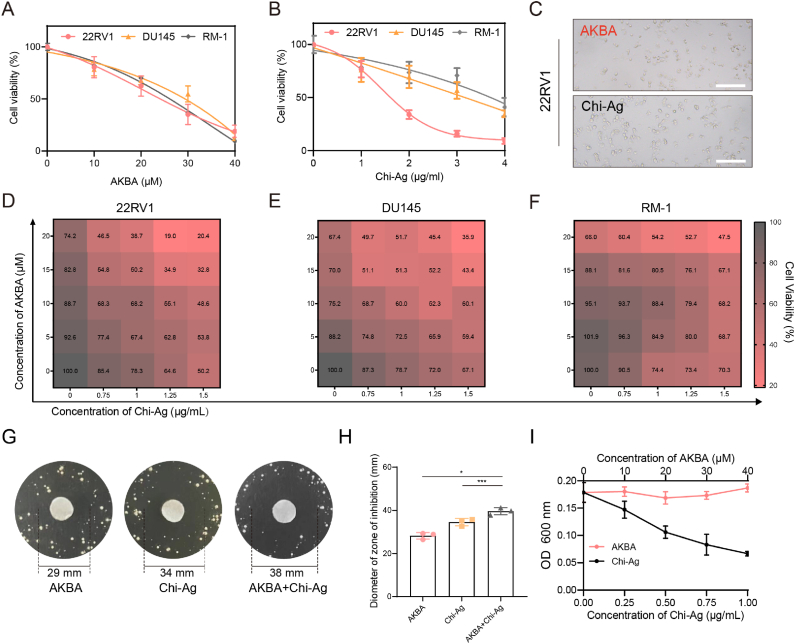
**AKBA synergizes with Chi-Ag NPs to kill PCa cells and *C.acnes in vitro*. (A)** Cell viability of 22RV1, DU145 and RM-1 cell lines at different concentrations of AKBA (0, 10, 20, 30, and 40 μM). **(B)** Cell viability of 22RV1, DU145 and RM-1 cell lines at different concentrations of Chi-Ag NPs (0, 1, 2, 3, and 4 μg/mL). **(C)** Light microscopy images showing the morphology of 22RV1 cells after 48 h of intervention with AKBA and Chi-Ag NPs. Scale bar: 100 μm. **(D**–**F)** Cell viability of 22RV1, DU145 and RM-1 cells with AKBA and Chi-Ag NPs co-treatment. **(G**–**H)** Photographs and the corresponding statistical graphs of inhibition zones of *C.acnes* with different treatments at the same concentration. **I.** The MIC Curves of Different Concentrations of AKBA (0, 10, 20, 30, and 40 μM) and Chi-Ag NPs (0.25, 0.5, 0.75, and 1 μg/mL).

AKBA is a recognized active compound with anti-PCa effects [24]. In our previous studies, the combination of Chi-Ag NPs with natural compounds demonstrated significant antibacterial and anti-tumor activity [10]. Here, we assessed the cytotoxicity of AKBA and Chi-Ag NPs against PCa cell lines. MTT assays indicated that both AKBA and Chi-Ag NPs induced tumor cell death in a dose-dependent manner. The IC_50_ values of AKBA after 48 h of treatment were 25.57, 28.55, and 26.42 μM for 22RV1, DU145, and RM-1 cells, respectively, while the IC_50_ values for Chi-Ag NPs were 1.864, 2.813, and 3.412 μg/mL for these cell lines (Fig. 3A–B). Both AKBA and Chi-Ag interventions result in cell death in22RV1 cells (Fig. 3C). Optimizing the concentrations of both agents revealed that a combination of 15 μM AKBA and 1 μg/mL Chi-Ag NPs resulted in a 50.2 % survival rate for 22RV1 cells, a dose significantly lower than the IC_50_ of AKBA alone (25.57 μM) (Fig. 3D). This synergistic effect was also observed in DU145 and RM-1 cells (Fig. 3E–F), suggesting that low-dose Chi-Ag NPs can enhance chemotherapy efficacy against PCa, consistent with previous findings.

Given that *C.acnes* can promote PCa invasion and metastasis, we further examined the antibacterial efficacy of Chi-Ag NPs. Agar diffusion and MIC assays were conducted to evaluate Chi-Ag NPs' capacity to inhibit *C.acnes*. The results demonstrated inhibition zone diameters of 29 mm and 34 mm for treatments with 100 μM AKBA (IC50 = 15 μM AKBA) and 10 μg/mL Chi-Ag NPs, respectively. Notably, combined treatment yielded an inhibition zone diameter of 38 mm, indicating a synergistic effect (Fig. 3G–H). The MIC assay revealed that AKBA alone, at concentrations below 40 μM, did not inhibit *C.acnes*, whereas the MIC of Chi-Ag NPs for *C.acnes* was only 0.63 μg/mL (Fig. 3I). These findings demonstrate that Chi-Ag NPs possess potent antibacterial activity even at low concentrations, providing a foundation for potential *in vivo* applications.

### 3.4 Preparation and characterization of SZTI01@APA NPs

**Scheme 1:**
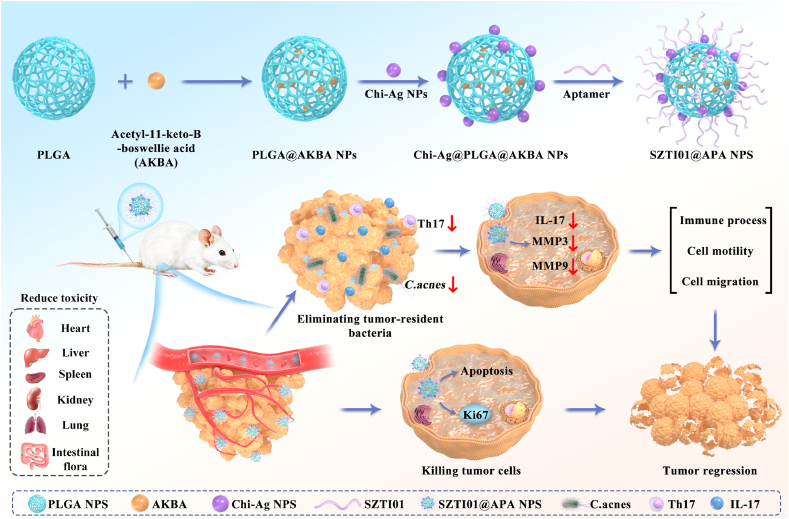
SZTI01@APA NPs for targeting synergistic treatment of PCa tumors and tumor-resident bacteria.

**Fig. 4:**
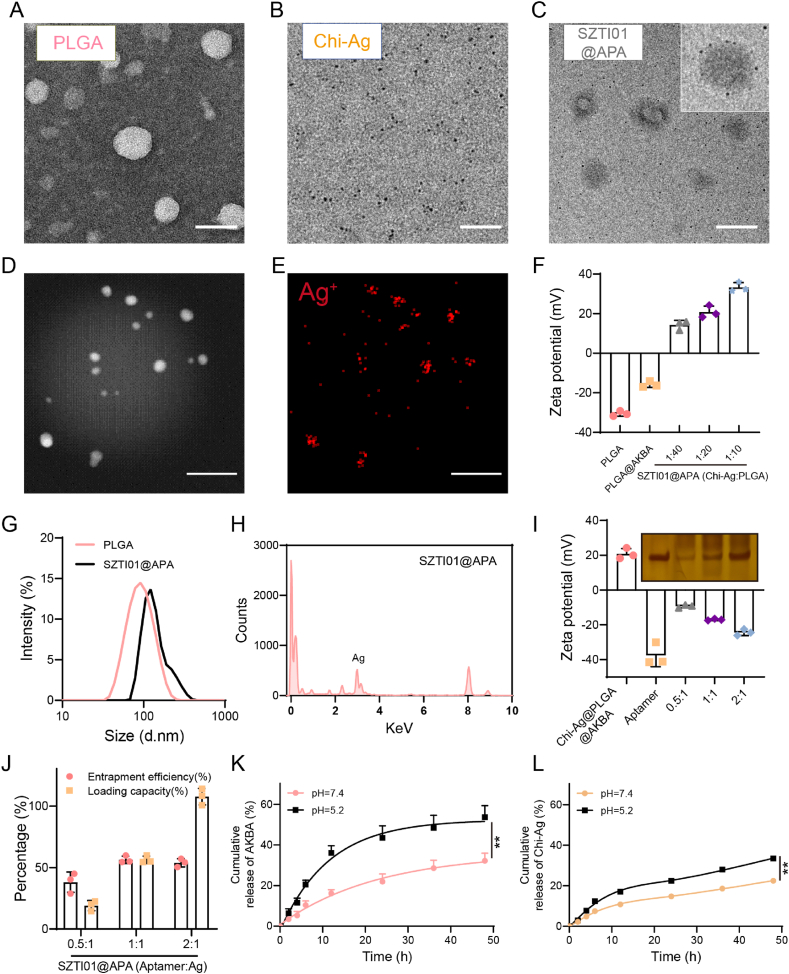
**Preparation and characterization of SZTI01@APA NPs. (A**–**C)** TEM images of PLGA, Chi-Ag NPs and SZTI01@APA NPs. Scale bar: 50 μm. **(D**–**E)** Mapping scan image of Chi-Ag NPs. Scale bar: 100 μm. **(F)** Zeta potential changes of PLGA NPs after adsorption of Chi-Ag NPs. **(G)** DLS data of PLGA and SZTI01@APA NPs. **(H)** EDS spectra of SZTI01@APA NPs. **(I)** Zeta potential changes and silver staining electrophoresis images after adsorption of different concentrations of SZTI01 with Chi-Ag@PLGA@AKBA NPs**. (J)** Entrapment efficiency and Loading capacity of SZTI01 at different concentrations. **(J)** Cumulative release of AKBA under different pH values. **(L)** Cumulative release of Chi-Ag NPs under different pH values.

To enhance the targeting ability and bioavailability, and reduce the side effects of AKBA and Chi-Ag NPs with known anti-tumor and anti**-*C.acnes*** efficacy, we employed nanotechnology to develop SZTI01@APA NPs for PCa treatment (Scheme 1). Consistent with previous studies, TEM images showed that unloaded PLGA NPs are spherical. After encapsulating AKBA and adsorbing Chi-Ag NPs and SZTI01 on the surface, SZTI01@APA NPs retained this spherical structure, with multiple visible spots on the NP surface (Fig. 4A–C). Meanwhile, to validate the spatial correlation between Ag^+^ ions and PLGA@AKBA NPs, we performed elemental mapping analysis of Ag^+^. The results demonstrated a significant overlap of PLGA@AKBA NPs and Ag^+^ signals in the mapping images, indicating that Ag^+^ was adsorbed onto the PLGA surface through electrostatic interactions (Fig. 4D–E). Chitosan, as a stabilizer for Ag NPs, contains surface hydroxyl (-OH) and amino (-NH_2_) groups that significantly stabilize Ag NPs through electrostatic interactions, preventing their aggregation [25]. Therefore, we further investigated the material stability of Chi-Ag NPs, and the results showed that the initially synthesized Chi-Ag NPs had a particle size of 29.3 nm and a surface charge of 38.4 mV. After storage in a 4 °C refrigerator for 1, 2, and 3 weeks, we analyzed the Chi-Ag NPs using a particle size and potential analyzer. The results revealed that after 1 week of storage, the particle size of Chi-Ag NPs increased while the surface potential decreased, and these changes stabilized after 2–3 weeks.(Fig. S1A–S1C). These minor changes in particle size and zeta potential may be associated with partial aggregation of the nanoparticles due to electrostatic shielding or intermolecular interactions of chitosan during storage [26]. The zeta potential of PLGA NPs and PLGA@AKBA NPs were −30.6 mV and −15.8 mV, respectively. Upon adsorption of positively charged Chi-Ag NPs, the surface charge of Chi-Ag@PLGA@AKBA NPs ranged from 14.3 mV to 33.3 mV, depending on the mass ratios (1:40 to 1:10) (Fig. 4F. The particle size of SZTI01@APA NPs increased from 90 nm to 110 nm after AKBA loading and Chi-Ag adsorption on the surface (Fig. 4G). EDS analysis confirmed the presence of Ag^+^ alongside C, H, and O, indicating successful Chi-Ag adsorption onto the nanosphere surface (Fig. 4H). Furthermore, LC-MS and UV-Vis spectroscopy revealed characteristic absorption peaks of AKBA and Chi-Ag NPs in the dialysis external solution and supernatants. The entrapment efficiency and loading capacity of AKBA were 75 % and 7.5 %, respectively (Fig. S2A–S2B). For PLGA and Chi-Ag NPs at 10:1, 20:1, and 40:1 ratios, Chi-Ag entrapment efficiencies were 39.1 %, 30.8 %, and 18.3 %, with corresponding loading capacities of 3.4 %, 1.4 %, and 0.4 % (Fig. S2C–S2D).

To further quantify SZTI01 adsorption, SDS-PAGE electrophoresis with silver staining was performed. The entrapment efficiency of SZTI01 was 56.4 % at a 1:1 mass ratio of SZTI01/Chi-Ag NPs, with only a slight increase to 57.1 % upon further SZTI01 addition, suggesting a saturation threshold. Surface potential measurements indicated negative potentials of −9.3 mV, −17.0 mV, and −24.3 mV for SZTI01-to-Chi-Ag ratios of 0.5:1, 1:1, and 2:2, respectively, attributable to the negative charge of the aptamer [27] (Fig. 4I–J).

Low pH is a significant characteristic of the tumor microenvironment, and the drug release level in this specific microenvironment is one of the key criteria for evaluating nano-systems [28]. Finally, we investigated the release profile of AKBA from SZTI01@APA NPs in simulated blood (pH 7.4) and lysosomal (pH 5.4) environments. Cumulative AKBA release was 32.1 % and 53.7 %, respectively (Fig. 4K), suggesting that the acidic lysosomal environment accelerates PLGA degradation. Additionally, under the same conditions, ICP-MS was used to measure the release rate of Ag^+^ under different pH conditions. The results showed that in microenvironments with pH 7.4 and 5.2, the cumulative release amounts of Ag^+^ were 22.4 % and 33.3 %, respectively (Fig. 4L). This property enables targeted AKBA and Chi-Ag NPs release within the acidic tumor cell lysosomes, thereby enhancing therapeutic efficacy while reducing systemic toxicity.

### 3.5 Targeting and penetration of SZTI01@APA NPs

**Fig. 5:**
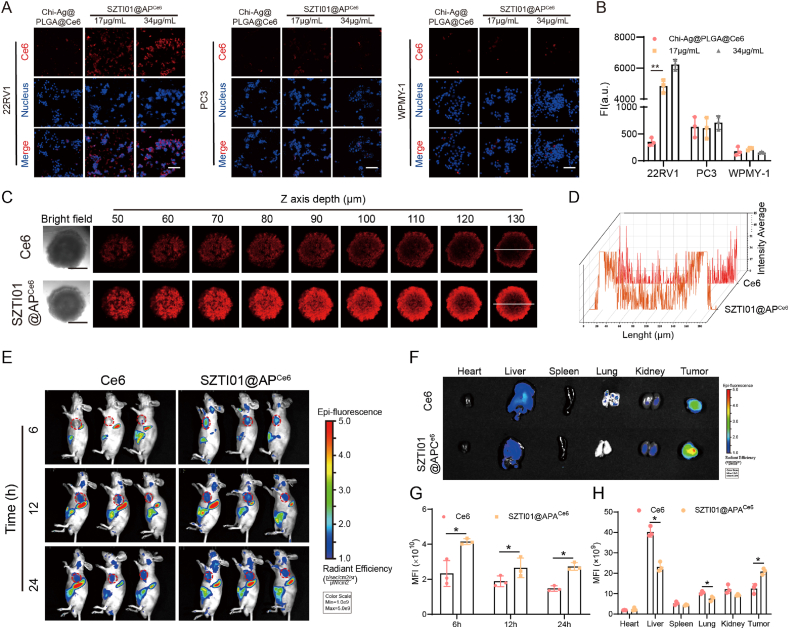
**Targeting and penetration of SZTI01@APA NPs. (A**–**B)** Fluorescence images and quantitative analysis of SZTI01@AP^Ce6^ NPs treated with 22RV1, PC3 and WPMY-1 cells. Scale bar: 50 μm. **(C)** Fluorescence images of 3D tumor spheres treatment with Ce6 and SZTI01@AP^Ce6^ NPs for 20 h. Scale bar: 100 μm. **(D)** Profiles show the fluorescence intensity along solid white lines on the sphere. **(E)** Fluorescence images of mice treated with different formulations at selected time points. **(F)***Ex vivo* fluorescence images of heart, liver, spleen, lung, kidney, and tumor at 24 h. **(G)** Quantitative analysis of mice after being treated with different formulations at desired time points. **(H)** Quantitative analysis of heart, liver, spleen, lung, kidney, and tumor at 24 h.

The superior targeting and penetration capability is a unique advantage of nanomedicine in antitumor applications, enabling targeted delivery to solid tumors and effectively increasing the local drug concentration at the tumor site [29]. For instance, leveraging the hypoxic and high-glutathione (GSH) tumor microenvironment characteristics, the developed microorganism-targeted nanodelivery system MCDP@Bif demonstrates tumor-selective release of doxorubicin (DOX) and CaO_2_, enabling synergistic chemo-chemodynamic therapy against breast cancer [30]. To assess the *in vitro* cellular uptake efficiency of SZTI01@APA NPs, we loaded the tracer Ce6 into the nanoparticles to prepare SZTI01@AP^Ce6^ NPs. Confocal microscopy images revealed the presence of fluorescence signals after incubating SZTI01@AP^Ce6^ NPs with WPMY-1 (normal prostate cells), 22RV1 (Prostate-specific membrane antigen [PSMA]-positive), and PC3 (PSMA-negative) cell lines for 4 h. SZTI01 modification significantly enhanced the uptake efficiency in 22RV1 cells, with fluorescence intensities increasing 4.9-fold and 6.1-fold at 17 μg/mL and 34 μg/mL concentrations, respectively, compared to Chi-Ag@PLGA@^Ce6^ NPs. However, no difference in fluorescence intensity was observed between Chi-Ag@PLGA@^Ce6^ NPs and SZTI01@AP^Ce6^ NPs in PC3 and WPMY-1 cells (Fig. 5A–B). To further demonstrate that the targeting ability of SZTI01@AP^Ce6^ is mediated by SZTI01, pre-treatment of 22RV1 cells with the SZTI01 aptamer resulted in a 7.83-fold attenuation of red fluorescence (Fig. S3). These findings demonstrate that SZTI01 modification effectively enhances uptake efficiency in 22RV1 cells via interaction between SZTI01 and the aptamer. Further evaluation of SZTI01@AP^Ce6^ NPs in a 3D tumor spheroid model of 22RV1 cells showed deeper and more intense red fluorescence along the Z-axis of the 3D tumor spheroid compared to free Ce6 (Fig. 5C–D), attributable to the positive surface charge conferred by the SZTI01 aptamer modification.

We also assessed the targeting ability and half-life of SZTI01@AP^Ce6^ NPs *in vivo*. As shown in Fig. S4A and S4B, the half-life of free Ce6 *in vivo* was 1.3 h, while Ce6 encapsulated in SZTI01@AP^Ce6^ NPs at the same dose extended to 2.6 h, representing a twofold increase. Additionally, fluorescence intensity at the tumor site displayed a general decrease over time, yet remained consistently higher in the SZTI01@AP^Ce6^ NPs group, with an average fluorescence intensity increase of 1.67-fold at 6, 12, and 24 h compared to the free Ce6 group (Fig. 5E and G). Furthermore, *ex vivo* fluorescence imaging 24 h post-administration demonstrated a significant 1.68-fold increase in tumor fluorescence signal for SZTI01@AP^Ce6^ NPs compared to the Ce6 group (Fig. 5F and H). These results indicate that SZTI01@AP^Ce6^ NPs enable precise targeting of PCa tumor tissue, reduce drug accumulation in normal organs, and effectively penetrate solid tumor tissues, laying a foundation for comprehensive and targeted *in vivo* therapy.

Aptamers, as single-stranded oligonucleotides, have attracted significant research attention due to their unique advantages, including scalable production, relatively low development costs, and superior tissue penetration capabilities [31]. However, their clinical translation remains limited by rapid clearance *in vivo* and nonspecific targeting. As a result, only one aptamer-based drug, Macugen®, has been approved by the FDA for local delivery in treating age-related macular degeneration. In this study, although the SZTI01 aptamer demonstrated considerable tumor-targeting ability, its *in vivo* pharmacokinetic properties and potential immunogenicity have not been fully elucidated, which restricts its clinical applicability. In subsequent research, various modification strategies can be employed to enhance its stability *in vivo*. For example, Wang et al. modified aptamers with polyphosphodiester backbones and polyethylene glycol (PEG) side chains, enabling tunability in aptamer size and structure while reducing nonspecific binding and extending blood retention time [32]. Therefore, further optimization and validation of the SZTI01 aptamer in preclinical studies are essential. Key future research directions should include extending its *in vivo* half-life, clarifying metabolic pathways, and evaluating immunogenicity.

### 3.6 Anti-tumor effect of SZTI01@APA NPs *in vitro*

**Fig. 6:**
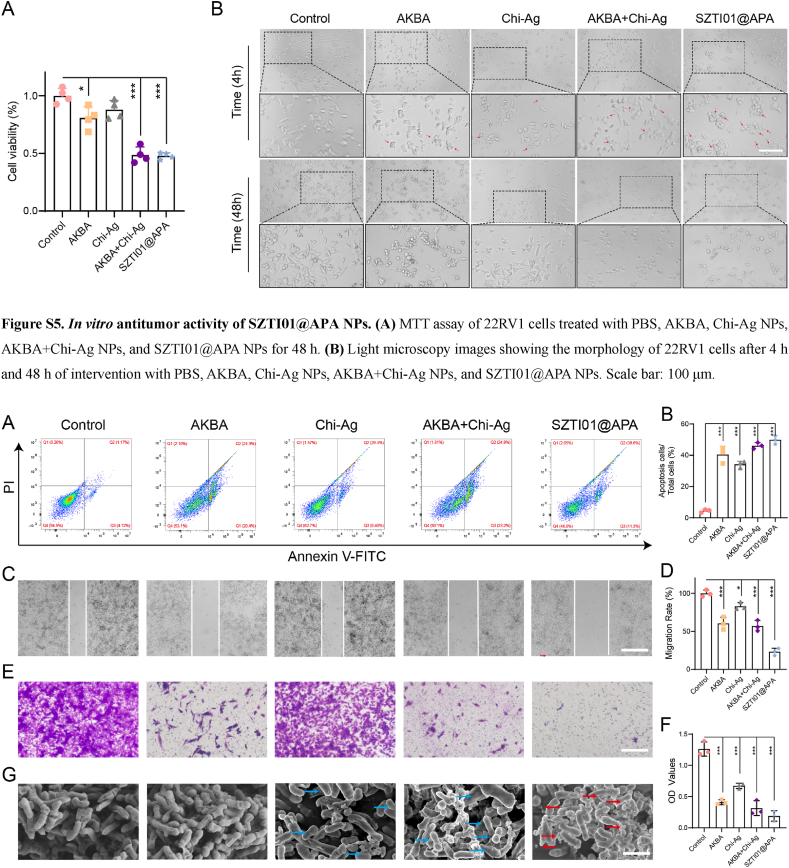
**Anti-tumor effect of SZTI01@APA NPs *in vitro*. (A)** Apoptosis rate and **(B)** statistical analysis of 22RV1 cells after 48 h of different treatments. **(C)** Representative images and **(D)** quantitative analysis of wound healing rates in scratch-wound assays of 22RV1 cells after 48 h of different treatments. Scale bar: 100 μm. **(E)** Typical photographs and **(F)** quantified percentage of invaded cells across the transwell membrane (pore size, 8.0 μm). Scale bar: 100 μm. **(G)** SEM images of *C.acnes* after different treatments. Scale bar: 1 μm.

To evaluate the cytotoxicity of SZTI01@APA NPs on 22RV1 cells, we performed an MTT assay, which revealed that all treated groups exhibited cell death, with cell survival rates of 80.8 %, 87.9 %, 48.6 %, and 47.8 % after 48 h of treatment, respectively (Fig. S5A–S5B). Morphological analysis showed the presence of apoptotic bodies (indicated by red arrows) in the AKBA, Chi-Ag NPs, AKBA + Chi-Ag NPs, and SZTI01@APA NPs groups at 4 h, with a notably higher number of apoptotic bodies in the AKBA + Chi-Ag NPs and SZTI01@APA NPs groups. Flow cytometry revealed apoptosis rates of 5.29 %, 40.44 %, 34.20 %, 46.15 %, and 49.86 % for PBS, AKBA, Chi-Ag NPs, AKBA + Chi-Ag NPs, and SZTI01@APA NPs, respectively (Fig. 6A–B). To further simulate *in vivo* anti-tumor treatment, we employed a 3D tumor spheroid model to assess the therapeutic effect of SZTI01@APA NPs. As shown in Fig. S6, the PBS group exhibited gradual tumor sphere growth from day 0 to day 7. In contrast, apoptosis was observed in the outer proliferative layer of the tumor spheres in the SZTI01@APA NPs group as early as day 1 post-treatment. By day 3, the tumor spheres began to disintegrate due to the structural breakdown of the outer layer. These findings suggest a synergistic effect between AKBA and Chi-Ag NPs, with nano-encapsulation preserving the cytotoxic effects of the original drugs. We further examined the impact of SZTI01@APA NPs on 22RV1 cell invasion and metastasis using wound healing and transwell assays. Compared to PBS, wound healing rates in cells treated with AKBA, Chi-Ag NPs, AKBA + Chi-Ag NPs, and SZTI01@APA NPs were reduced by 39.6 %, 26.9 %, 42.9 %, and 76.9 %, respectively (Fig. 6C–D). Similarly, all treatments reduced the number of invading 22RV1 cells in the transwell assay. The OD values at 570 nm were 0.41, 0.67, 0.31, and 0.19 for AKBA, Chi-Ag NPs, AKBA + Chi-Ag NPs, and SZTI01@APA NPs, respectively (Fig. 6E–F). Finally, we utilized SEM microscopy to observe the effects of different treatments on *C.acnes* bacteria. In the PBS and AKBA groups, bacterial membranes remained intact with smooth edges, clear textures on the cell walls, and a regular rod shape. In contrast, the Chi-Ag NPs, AKBA + Chi-Ag NPs, and SZTI01@APA NPs groups exhibited Chi-Ag NP (blue arrows) and SZTI01@APA NP (red arrows) coverage on the bacterial membranes, with varying degrees of surface irregularities and structural damage, including irregular shapes, round structures, and areas of bacterial debris fusion (Fig. 6G). These results indicate that SZTI01@APA NPs inhibit 22RV1 cell proliferation and invasion *in vitro*, exert strong bactericidal effects on tumor-associated bacteria, and significantly induce apoptosis in both 2D and 3D tumor models.

### 3.7 *In vivo* anti-tumor effect of SZTI01@APA NPs

**Fig. 7:**
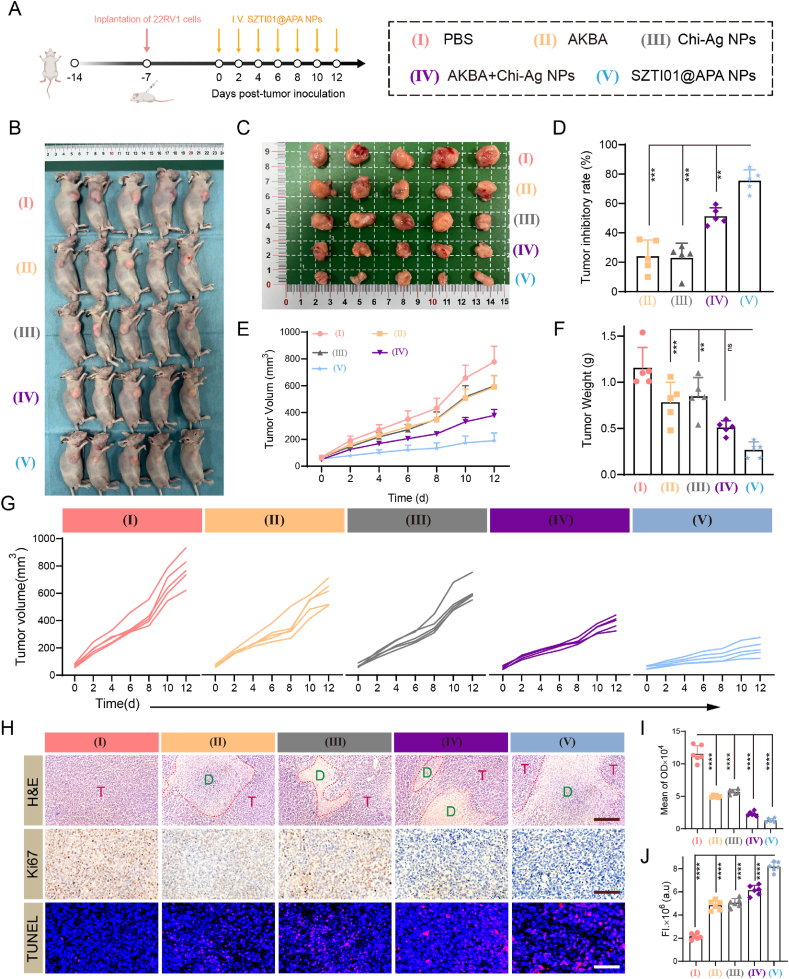
***In vivo* anti-tumor effect of SZTI01@APA NPs. (A)** Schematic of the animal experimental design. **(B**–**C)** Representative images of 22RV1 tumor-bearing mice and excised tumors following different treatments (a: PBS; b: AKBA; c: Chi-Ag NPs; d: AKBA + Chi-Ag NPs; e: SZTI01@APA NPs). **(D)** The tumor inhibitory rate of mice with different treatments. **(E**–**F)** Tumor volume and weight in 22RV1 tumor-bearing mice under different treatment conditions. **(G)** Tumor growth curves of mice in different groups. **(H)** H&E staining, Ki67 and TUNEL expression of tumor tissues with various treatments. Scale bar: 200 μm. **(I**–**J)** Quantification of Ki67 and TUNEL expression in tumor tissues across treatment groups.

Building on the promising *in vitro* antitumor effects of SZTI01@APA NPs, we further assessed their efficacy in a 22RV1 subcutaneous xenograft tumor model *in vivo*. The experimental design (Fig. 7A) involved monitoring tumor volume and body weight changes in nude mice over the study period, with tumor tissues collected at the study's conclusion. As shown in Fig. 7B–G, free drug treatments had limited effects on tumor growth, whereas drug combinations exhibited synergistic antitumor effects. The addition of the targeting nucleic acid aptamer SZTI01 further enhanced efficacy, achieving tumor inhibition rates (TIR) of 24.1 % (AKBA), 23.0 % (Chi-Ag NPs), 51.3 % (AKBA + Chi-Ag NPs), and 75.5 % (SZTI01@APA NPs). Tumor weight analysis at the end of the treatment confirmed that the SZTI01@APA NPs group had superior therapeutic effects, with reductions in tumor weight of 32.2 %, 26.6 %, 55.9 %, and 76.8 % for PBS, AKBA, Chi-Ag NPs, and AKBA + Chi-Ag NPs groups, respectively. These findings indicate that SZTI01@APA NPs, through the combination of drugs and aptamer targeting, deliver potent antitumor efficacy, supporting their potential in synergistic therapy and clinical applications of nano-delivery systems.

To further explore the mechanisms underlying the antitumor effect of SZTI01@APA NPs, we analyzed histopathological and molecular markers in tumor tissues. Tumor sections from the SZTI01@APA NPs group showed extensive areas of necrosis (dotted areas) (Fig. 7H). Ki67, a proliferation-associated antigen and important prognostic marker for PCa [33], is strongly associated with higher Gleason scores in PCa [34]. Ki67 staining results showed that SZTI01@APA NPs significantly reduced Ki67 expression in tumor tissues, with reductions of 88.8 %, 74.1 %, 77.3 %, and 44.1 % compared to the PBS, AKBA, Chi-Ag NPs, and AKBA + Chi-Ag NPs groups, respectively (Fig. 7I). Additionally, TUNEL staining of tumor tissues from SZTI01@APA NPs-treated mice revealed a marked increase in red fluorescence, indicating a significant rise in TUNEL-positive apoptotic cells. Compared to the PBS group, the apoptosis rate in the SZTI01@APA NPs group increased by 3.80-fold (Fig. 7J). These results suggest that the potent antitumor activity of SZTI01@APA NPs in PCa may be attributed to their ability to inhibit tumor proliferation and induce apoptosis in tumor tissue.

### 3.8 SZTI01@APA NPs reduce Th17 cell differentiation by inhibiting *C.acnes* activity

**Fig. 8:**
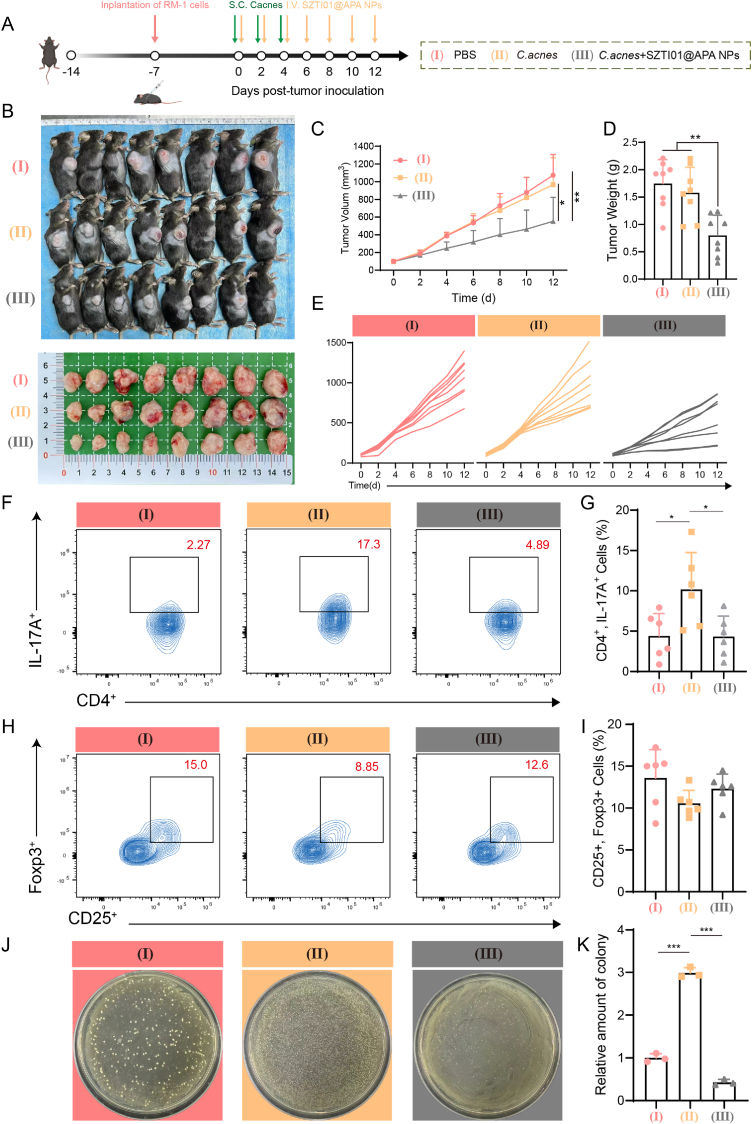
**SZTI01@APA NPs reduce Th17 cell differentiation by inhibiting *C.acnes* activity. (A)** Diagram of the animal experiment. **(B)** Images of RM-1 tumor-bearing mice and tumors following different treatments (a: PBS; b: *C.acnes*; c: *C.acnes* + SZTI01@APA NPs). **(C**–**D)** Tumor volume and weight of RM-1 tumor-bearing mice under different treatments. **(E)** Tumor growth curves of mice in different groups. **(F**–**I)** Representative flow cytometry and quantitative analysis of the Th17 and Treg cells in tumor tissues at the end of therapy. **(J**–**K)** Images and quantitative analysis of tumor tissue bacterial dilution plates with different treatments.

Building on the potent *in vivo* anti-tumor and anti-metastatic effects of SZTI01@APA NPs, we established a *C.acnes*-infiltrated subcutaneous tumor model using RM-1 cells in BALB/c mice to further explore the therapeutic efficacy of SZTI01@APA NPs. The experimental setup is outlined in Fig. 8A. Tumor volume and body weight were monitored throughout the study, and tumor tissues were collected from each group at the experiment's conclusion. As shown in Fig. 8B–E, while *C.acnes* infiltration did not significantly impact tumor growth compared to the PBS group, treatment with SZTI01@APA NPs led to notable tumor inhibition, with a tumor suppression rate of 48.4 % and a reduction in tumor weight by 54.3 % compared to the PBS group. These results suggest that SZTI01@APA NPs retain their tumor-inhibiting effects despite *C.acnes* intervention. Previous research has demonstrated that *Propionibacterium acnes* can modulate immune cell infiltration in tumors. To assess this, we analyzed immune cell infiltration in tumor tissues following SZTI01@APA NPs treatment via flow cytometry. In the PBS, *C.acnes,* and *C.acnes* + SZTI01@APA NPs groups, the proportions of Th17 cells (CD4^+^, IL-17A^+^) were 4.4 %, 10.1 %, and 4.3 %, respectively. *C.acnes* infiltration significantly increased Th17 cell differentiation, with a 2.2-fold increase in the *C.acnes* group compared to PBS. SZTI01@APA NPs treatment markedly reduced Th17 cell proportions, decreasing it by 57.5 % compared to the *C.acnes* group. Th17 cells, involved in cancer-related inflammation, secrete factors such as IL-17A, IL-17F, and IL-22, which promote PCa cell viability, migration, and invasion [19]. SZTI01@APA NPs effectively countered the Th17 cell increase induced by *C.acnes,* potentially contributing to the observed inhibition of PCa progression and metastasis. Additionally, we analyzed Treg cell (CD25^+^, Foxp3^+^) expression in tumor tissues. The proportions of Treg cells in the PBS, *C.acnes,* and *C.acnes* + SZTI01@APA NPs groups were 13.5 %, 10.6 %, and 12.3 %, respectively. Compared to the PBS group, both the C.acnes group and the C.acnes + SZTI01@APA NPs group showed a reduction in the proportion of Treg cell infiltration, but the difference was not statistically significant (Fig. 8F–I). Finally, we evaluated the effect of SZTI01@APA NPs on tumor-resident bacterial activity by culturing bacteria from tumor tissues. Numerous bacterial colonies were observed in the *C.acnes* group, with quantitative analysis revealing a 2.9-fold increase in colony numbers compared to PBS. In the SZTI01@APA NPs-treated group, the number of bacterial colonies decreased by 87.1 % compared to the *C.acnes* infiltration group (Fig. 8J–K). Collectively, these results indicate that *C.acnes* infiltration disrupts the Th17/Treg balance in tumor tissues, increasing Th17 cell infiltration in the tumor microenvironment. SZTI01@APA NPs effectively eliminated *C.acnes* from tumors, mitigating this effect and restoring immune balance.

### 3.9 SZTI01@APA NPs down-regulating IL-17 signal pathway in *C.acnes*-infiltrated subcutaneous tumor model

**Fig. 9:**
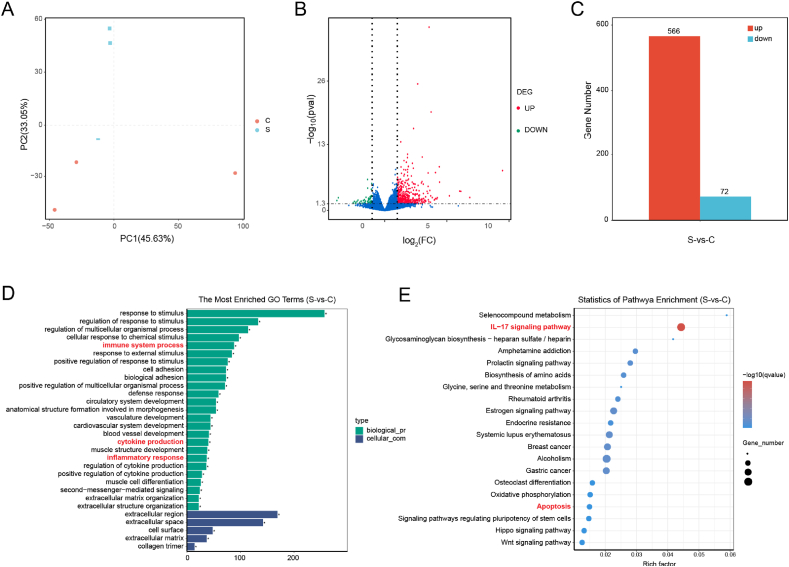
**SZTI01@APA NPs down-regulating IL-17 signal pathway in *C.acnes*-infiltrated subcutaneous tumor model. (A)** Principal component analysis (PCA) of differentially expressed genes of the transcriptomics profile of cells with *C.acnes* (c) or *C.acnes* + SZTI01@APA NPs (s). **(B)** Volcano plot and **(C)** Bar chart of gene expression changes in tumor tissues post-SZTI01@APA NPs treatment, showing upregulated and downregulated genes. **(D)** GO enrichment analysis of differentially expressed genes involved in biological processes. **(E)** KEGG enrichment analysis of differentially expressed genes in different signaling pathways. (c: *C.acnes* group; z: *C.acnes* + SZTI01@APA NPs group).

To investigate the *in vivo* therapeutic mechanism of SZTI01@APA NPs against PCa, we conducted a transcriptomic analysis to examine gene expression changes following treatment. PCA plot results showed good reproducibility between the two sample groups in three-dimensional space (Fig. 9A).Based on the criterion of p < 0.05, in the *C.acnes* infiltration model treated with SZTI01@APA NPs, 566 genes were upregulated and 72 genes were downregulated (Fig. 9B and C). Gene Ontology (GO) enrichment analysis revealed that the DEGs regulated by SZTI01@APA NPs were primarily associated with biological processes such as immune system process, cytokine production and inflammatory response. Additionally, the cellular components involved in regulating these DEGs included the extracellular region, extracellular space, cell surface, extracellular matrix, and collagen trimer (Fig. 9D). Based on the GO enrichment analysis, it can be inferred that SZTI01@APA NPs may inhibit *C.acnes*-infiltrated subcutaneous tumor model growth by modulating extracellular immune-related pathways. Further integration of the differentially expressed genes with the KEGG database highlighted the IL-17 signaling pathway as notably enriched among the downregulated pathways. Additionally, KEGG enrichment analysis revealed that the DEGs were significantly enriched in the apoptosis signaling pathway, suggesting that SZTI01@APA NPs may inhibit PCa progression by inducing tumor cell apoptosis (Fig. 9E). IL-17, predominantly secreted by Th17 cells, is a pro-inflammatory cytokine that stimulates the production of other inflammatory cytokines and chemokines [35]. The IL-17 pathway is known to promote tumor invasion and metastasis by upregulating MMPs [36]. For instance, Zhang et al. reported that inhibiting IL-17 receptor expression reduced MMP3 and MMP7 levels in tumor tissues in a PCa mouse model, thereby decreasing extracellular matrix degradation and tumor infiltration [37]. Previously, our research results showed that *C.acnes* induced the expression of MMP3 and MMP9, which promoted the invasion and metastasis of PCa, and the colonization of *C.acnes* in tumor tissues could be inhibited. We speculated that SZTI01@APA NPs downregulated the IL-17 pathway by inhibiting the colonization of C.acnes, thereby suppressing the expression of MMP3 and MMP9, and reducing the invasion and metastasis potential of PCa.

### 3.10 SZTI01@APA NPs reduce the abundance of *Firmicutes* and *Actinobacteria* while maintaining the balance of the gut microbiota

**Fig. 10:**
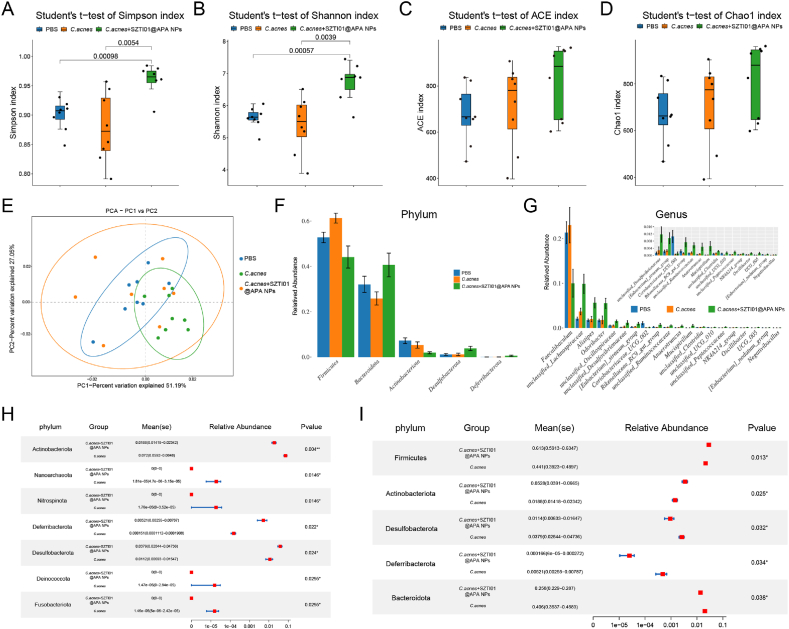
**SZTI01@APA NPs maintain the diversity of the gut microbiota. (A**–**D)** Analysis of alpha diversity coefficient of intestinal flora, Simpson index, Shannon index, Ace index and Chao index. **(E)** Principal component analysis (PCA) was performed for beta diversity analysis to study the overall structural changes in the intestinal flora after SZTI01@APA NPs intervention. **(F)** Relative abundance analysis at the phylum level for bacterial communities in specified samples. **(G)** Relative abundance analysis at the genus level for bacterial communities in specified samples. **(H)** Comparative analysis of phylum-level bacterial abundance between the *C.acnes* + SZTI01@APA NPs group and the PBS group. **(I)** Comparative analysis of phylum-level bacterial abundance between the *C.acnes* + SZTI01@APA NPs group and the *C.acnes* group.

Antibiotic treatments are commonly used to combat bacterial infections but can disrupt the gut microbiota, resulting in reduced diversity and richness, which may lead to unexpected health complications [38]. To assess the impact of SZTI01@APA NPs on gut microbiota, fecal samples were collected from RM-1 cell BALB/c mice with subcutaneous tumors and *C.acnes* infiltration, followed by 16S rDNA sequencing. Alpha diversity was measured using the Simpson, Shannon, ACE, and Chao indices. While no significant differences in richness and diversity were observed between the PBS and *C.acnes* groups, SZTI01@APA NPs treatment led to a significant increase in both metrics compared to PBS and *C.acnes* groups (Fig. 10A–D). This suggests that the targeted action of SZTI01@APA NPs helps preserve gut microbiota diversity despite treatment. Beta diversity analysis, using PCA, demonstrated differences in microbial community composition among the groups, with the *C.acnes* + SZTI01@APA NPs group showing a reduced structural shift compared to the *C.acnes* group (Fig. 10E).

Existing studies suggest that gut microbiota can promote castration-resistant PCa (CRPC) through androgen synthesis pathways [39]. Additionally, the abundance of Firmicutes has been positively associated with serum testosterone levels in PCa patients [40]. We further analyzed the effects of SZTI01@APA NPs on gut microbiota composition. SZTI01@APA NPs treatment reduced the abundance of *Firmicutes* by 16.5 % and 28.1 % and *Actinobacteria* by 73.8 % and 64.5 % compared to PBS and *C.acnes* groups, respectively, while increasing *Bacteroidetes* by 26.7 % and 56.9 % (Fig. 10F). At the genus level, *Faecalibaculum*, *Lachnospiraceae*, *Alistipes*, *Odoribacter*, and *Oscillospiraceae* were the most abundant. The treatment reduced *Faecalibaculum* by 53.0 % and 56.4 % compared to PBS and *C.acnes* groups, while increasing *Lachnospiraceae*, *Alistipes*, *Odoribacter*, and *Oscillospiraceae* (Fig. 10G). Statistical analysis showed that SZTI01@APA NPs significantly altered the abundance of seven microbial phyla, including *Actinobacteriota*, *Nanoarchaeota*, *Nitrospinota*, *Deferribacterota*, *Desulfobacterota*, *Deinococcota*, and *Fusobacteriota*, compared to PBS (Fig. 10H). When compared to the *C.acnes* group, five phyla—*Firmicutes*, *Actinobacteriota*, *Desulfobacterota*, *Deferribacterota*, and *Bacteroidota*—were significantly altered (Fig. 10I). In conclusion, SZTI01@APA NPs effectively maintain gut microbiota stability, unlike conventional antibiotics that often decrease microbial diversity and abundance. SZTI01@APA NPs may play a role in inhibiting PCa progression by modulating gut microbiota composition, though further studies are required to elucidate the exact mechanisms. Meanwhile, at the phylum level, we observed that administration of SZTI01@APA NPs downregulated the abundance of *Firmicutes*. Notably, according to a study by Pernigoni et al., three androgen-synthesis-promoting bacterial species all belong to the *Firmicutes* phylum [39]. Therefore, the reduction in *Firmicutes* induced by SZTI01@APA NPs may represent an additional mechanism through which it inhibits the development of CRPC. Further exploration of the relationship between this pathway and alterations in androgen levels associated with CRPC will constitute a novel direction for future research on the anti-prostate cancer effects of SZTI01@APA NPs. Furthermore, existing studies have reported that gut microbiota can modulate responses to tumor chemotherapy and immunotherapy. For example, research has shown that butyrate, a metabolite derived from gut microbes, can enhance the efficacy of oxaliplatin by regulating the function of CD8^+^ T cells within the tumor microenvironment [41]. This study preliminarily reveals the impact of SZTI01@APA NPs on the abundance and composition of the gut microbiota in mice. In subsequent research, one key mechanism to be further explored will be the integration of tumor-resident bacteria with gut microbes and their associated metabolites to collaboratively elucidate their regulatory effects on the tumor immune microenvironment.

### 3.11 Toxicity of SZTI01@APA NPs

Ensuring the biosafety of nanoparticle drugs is crucial for their clinical application [42]. The safety profile of Ag NPs poses a major challenge to their clinical translation for *in vivo* drug delivery. Some studies have reported that prolonged administration of Ag NPs can lead to alveolar inflammation and degeneration of neurons in the brain [43,44]. One important strategy for improving the biosafety of Ag NPs is to use chitosan coating, which can slow the release rate of Ag NPs and prevent their direct contact with tissues [45]. Therefore, we further investigated the safety of both Chi-Ag NPs and the composite nanoparticle system SZTI01@APA NPs. First, we evaluated the *in vitro* biocompatibility and toxicity of SZTI01@APA NPs in normal cells. As shown in Fig. S7A–S7B, SZTI01@APA NPs had a hemolysis rate of less than 5 % at concentrations from 0 to 200 μg/mL. Additionally, a coagulation assay showed that plasma hemolysis rates remained below 10 % after SZTI01@APA NP treatment (Fig. S7C). Cytotoxicity assays on normal cell lines demonstrated that SZTI01@APA NPs exhibited minimal cytotoxicity at concentrations higher than those applied in this study (AKBA and Chi-Ag concentrations of 15 μM and 1 μg/mL, respectively), with negligible effects on WPMY-1, VSM, HUVECs, HK2, and LO-2 cells (Fig. S7D). To further assess *in vivo* toxicity, we examined SZTI01@APA NP-treated mice through histological analysis, blood tests, and liver and kidney function tests. No pathological changes were observed in major organs following SZTI01@APA NP treatment compared to the PBS group, as shown by H&E staining (Fig. S8A). Blood routine analyses and liver function tests indicated that aspartate aminotransferase (AST) levels increased after modeling and treatment with AKBA, Chi-Ag NPs, and AKBA + Chi-Ag NPs. However, SZTI01@APA NP treatment reduced AST levels by 29.2 %, 36.0 %, and 16.3 % compared to AKBA, Chi-Ag NPs, and AKBA + Chi-Ag NPs groups, respectively (Fig. S8B). These findings suggest that the SZTI01@APA nanocomplex provides high biosafety, minimizing potential liver-related side effects associated with the long-term use of individual drugs.

Zebrafish, due to their sensitivity to environmental changes, are an ideal model for drug safety assessment [46]. We exposed zebrafish embryos to varying concentrations of SZTI01@APA NPs and found increased mortality rates at 24, 48, and 96 h post-exposure at 100 and 200 μg/mL (Fig. S9A–S9D). At 48 h, a slight increase in average heart rate was observed in the SZTI01@APA NP-treated group, along with a slight developmental delay in average body length. Collectively, these results confirm the acceptable safety profile of SZTI01@APA NPs for *in vivo* use, supporting their potential for clinical applications. Although the results of this study demonstrate a certain degree of biosafety of SZTI01@APA NPs, the duration of administration in this animal experiment was insufficient to adequately evaluate its chronic toxicity. Follow-up studies should employ longer-term *in vivo* administration to clarify the chronic toxicity profile of SZTI01@APA NPs. Furthermore, a more detailed elucidation of the safety of Chi-Ag NPs could be achieved through investigations into their biodistribution and drug metabolism. Conducting these studies is of great significance for a thorough assessment of the clinical translation potential of both Chi-Ag NPs and SZTI01@APA NPs.

## 4 Conclusion

This study developed a novel and effective nanocomplex for treating PCa by targeting both tumor cells and tumor-resident bacteria. The nanodrug inhibited PCa growth and metastasis through three main mechanisms: (1) The nanoparticle drug formulation, modified with the aptamer SZTI01, significantly enhanced tumor-targeting and penetration at the tumor site; (2) SZTI01@APA NPs, with high accumulation at the target site, inhibited tumor growth and migration by downregulating pathways involving IL-17, MMP3, and MMP9; and (3) Eradication of *C.acnes* in tumor tissues by SZTI01@APA NPs significantly reduced Th17 cell infiltration within the local PCa tumor environment. Nevertheless, this study has several limitations. While Chi-Ag nanoparticles exhibit significantly lower physiological toxicity than free Ag^+^ ions, these results have thus far only been confirmed in *in vitro* and preclinical studies [47]. Further large-scale clinical trials with long-term follow-up are needed to comprehensively assess their safety and therapeutic efficacy. Additionally, the clinical translation of aptamers faces persistent challenges, including off-target effects and potential delayed toxicity *in vivo* [48]*.* Consequently, aptamer screening and optimization constitute critical priorities for future investigation in this field. Ultimately, although our findings demonstrate the therapeutic promise of tumor-colonizing bacteria in PCa, resolving microbiome-host interaction variability remains a fundamental challenge that must be systematically addressed for successful clinical translation.

## CRediT authorship contribution statement

**Bo Zou:** Writing – original draft, Software, Data curation. **Xuefei Tian:** Writing – review & editing, Methodology, Investigation. **Ruisong Gao:** Project administration, Formal analysis. **Hongping Long:** Software, Project administration. **Yan Long:** Data curation, Conceptualization. **Bin Liu:** Writing – review & editing, Visualization, Methodology. **Qing Zhou:** Writing – review & editing, Visualization, Supervision, Resources.

## Funding

This work was supported by the 10.13039/501100001809National Natural Science Foundation of China [No. 82074450, No. 82374460, No. 82505602 and No. U20A20408]; Project on Advantages of Traditional Chinese Medicine in Treating Specific Diseases (Enhancement of Clinical Evidence-Based Capacity) [czxm-kyb-2025001].

## Declaration of competing interest

The authors declare the following financial interests/personal relationships which may be considered as potential competing interests: Qing Zhou and Bin Liu reports financial support was provided by The First Hospital of 10.13039/501100014978Hunan University of Chinese Medicine. Qing Zhou reports a relationship with The First Hospital of Hunan University of Chinese Medicine that includes: employment. If there are other authors, they declare that they have no known competing financial interests or personal relationships that could have appeared to influence the work reported in this paper.

## Data Availability

No data was used for the research described in the article.
