# Supplementary material for: 3-acetyl-11-keto-β-boswellic acid and chitosan-Ag nanoparticles for synergistic tumor-resident bacteria mediated prostate cancer therapy
Source: Mater Today Bio. 2025 Oct 16;35:102374. doi: 10.1016/j.mtbio.2025.102374 (PMC12554191; doi:10.1016/j.mtbio.2025.102374)
Supplement: Multimedia component 1 [file mmc1.docx]

#### **Supplementary material**

**3-acetyl-11-keto-*β*-boswellic acid and chitosan-Ag nanoparticles for synergistic tumor-resident bacteria mediated prostate cancer therapy**

Bo Zou^1^, Xuefei Tian^2^, Ruisong Gao^1^, Hongping Long^1^, Yan Long^1^, Bin Liu^3^*, Qing Zhou^1^**

^1^ The First Hospital of Hunan University of Chinese Medicine, Changsha 410007, Hunan, China

^2^ Hunan University of Chinese Medicine, Changsha 410208, Hunan, China,

^3^ College of Biology of Hunan University, Changsha 410208, Hunan, China

Corresponding authors: 310094@hnucm.edu.cn (Q Zhou); E-mail: binliu2001@hotmail.com (B Liu)


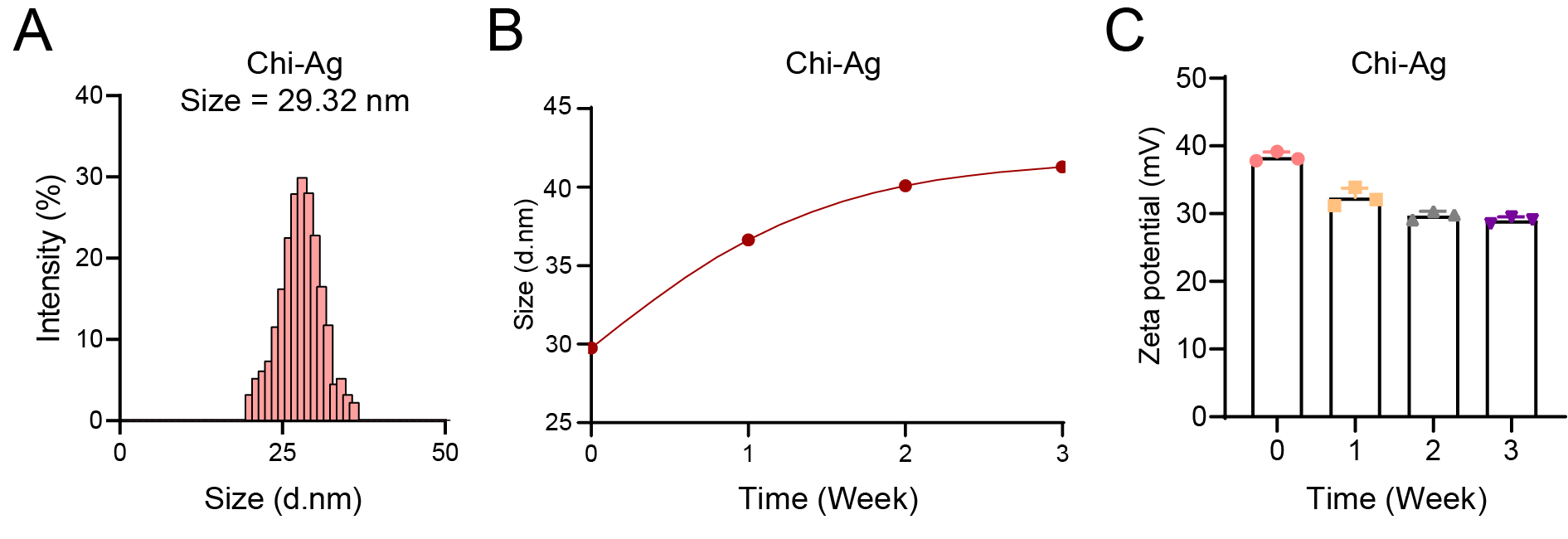


**Fig S1. Characterization of Chi-Ag NPs.** (A) DLS data of Chi-Ag NPs. (B) Changes in particle size of Chi-Ag NPs over 1–3 weeks after synthesis. (C) Changes in surface charge of Chi-Ag NPs over 1–3 weeks after synthesis.


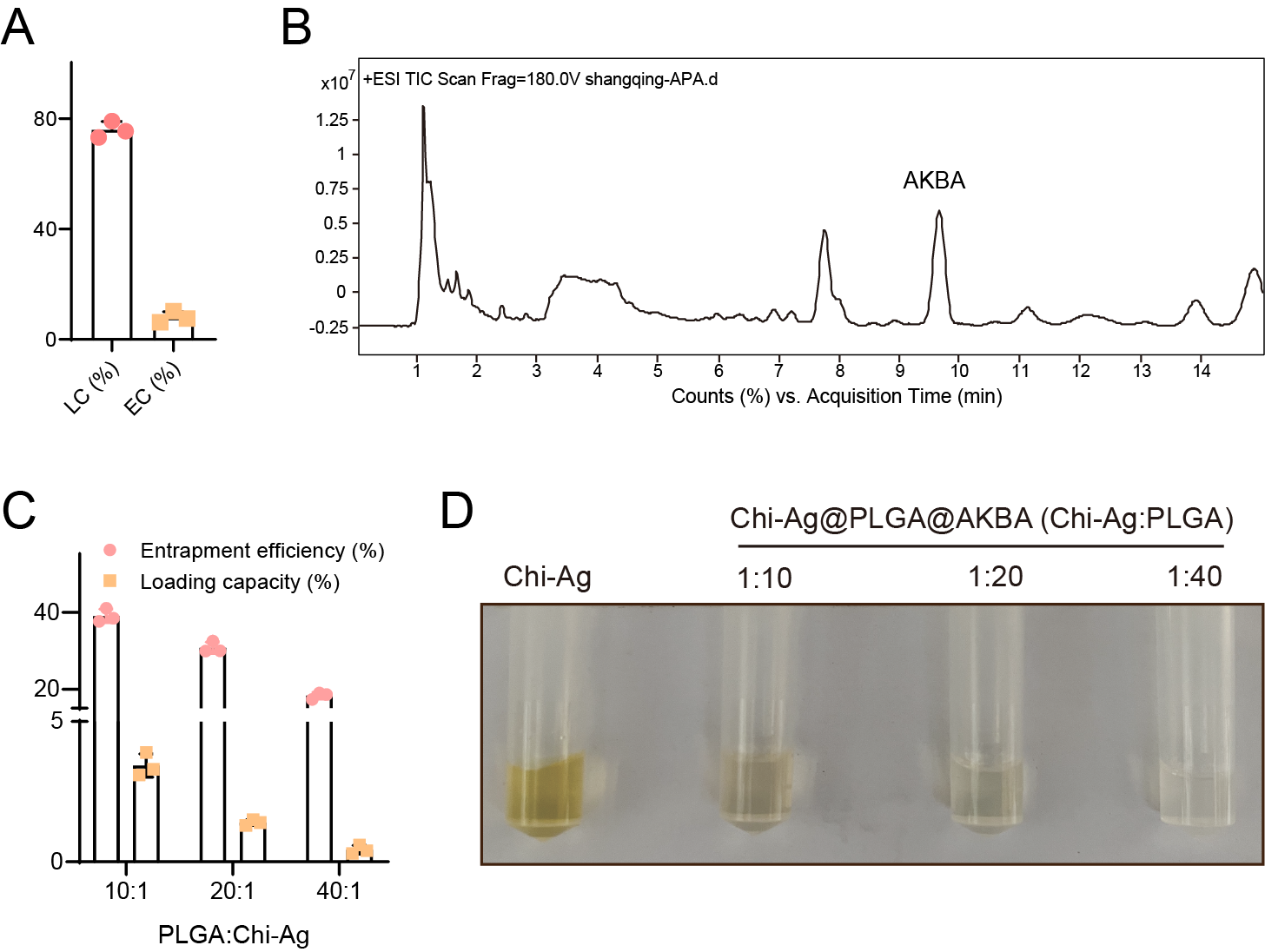


**Figure S2. Entrapment efficiency and loading capacity of AKBA and Chi-Ag NPs. (A)** Entrapment efficiency and loading capacity of AKBA. **(B)** LC-MS spectrum of AKBA in dialysate and centrifuged supernatants**. (C)** Entrapment efficiency and Loading capacity of Chi-Ag NPs. **(D)** Images of Chi-Ag NPs and Chi-Ag@PLGA@AKBA NPs at room temperature for 4 h.


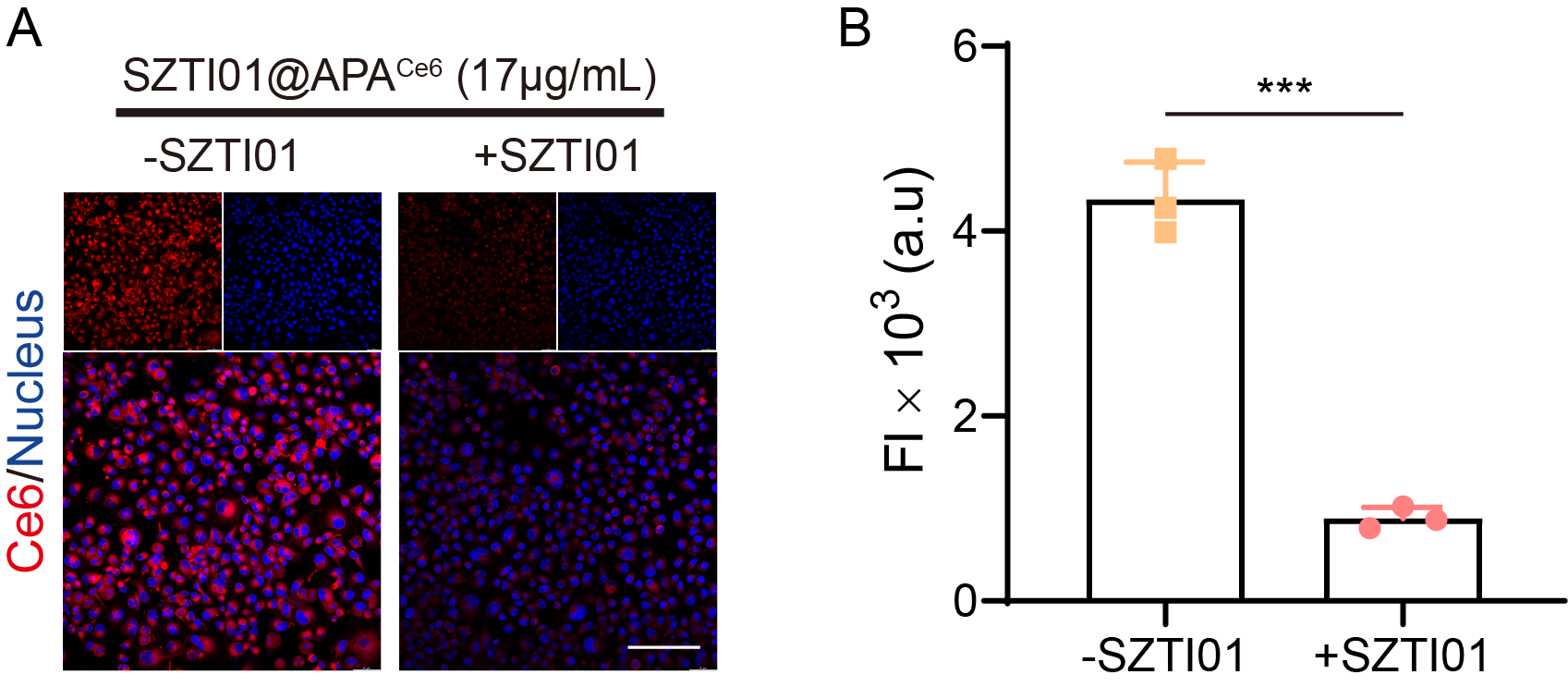


**Fig S3.** **(A)** CLSM images of cellular uptake of SZTI01@AP^Ce6^ NPs (concentration of Ce6 is 10 μg/mL) in 22RV1 with/without SZTI01 and **(B)** fluorescence intensity analysis. Scale bar: 100 μm.


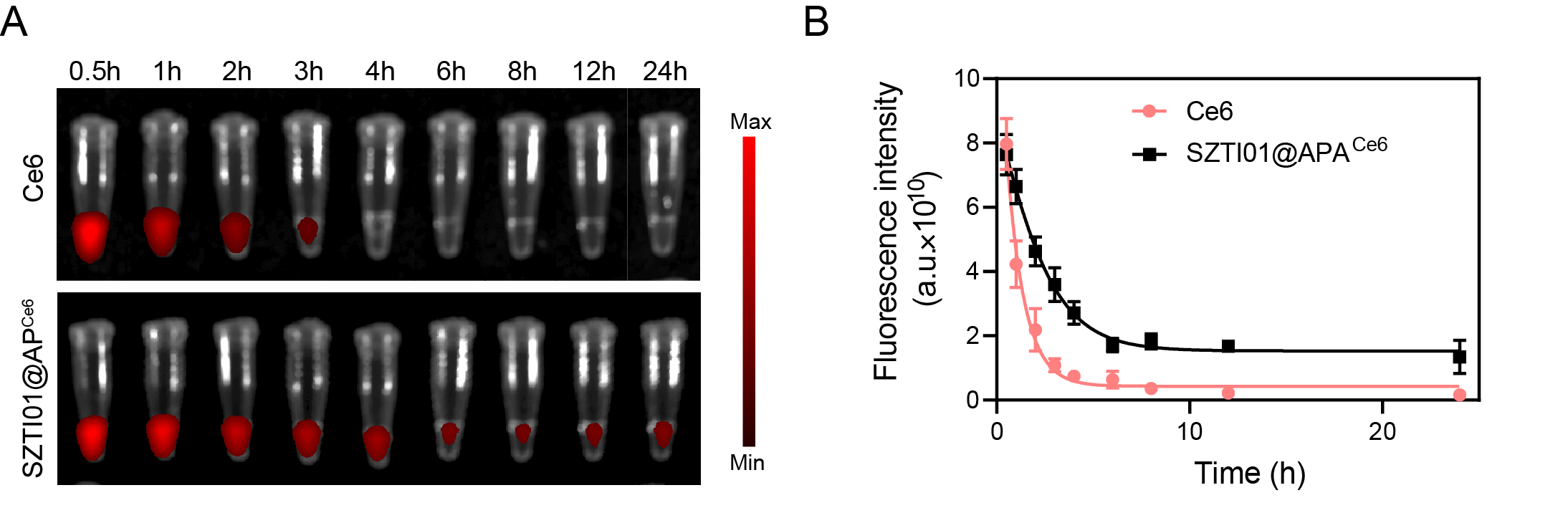


**Figure S4. The half-life of SZTI01@AP^Ce6^ NPs *in vivo.*** **(A)** Blood fluorescence intensity of Ce6 and SZTI01@AP^Ce6^ NPs over time. **(B)** Pharmacokinetic curves of Ce6 and SZTI01@AP^Ce6^ NPs after a single intravenous injection.


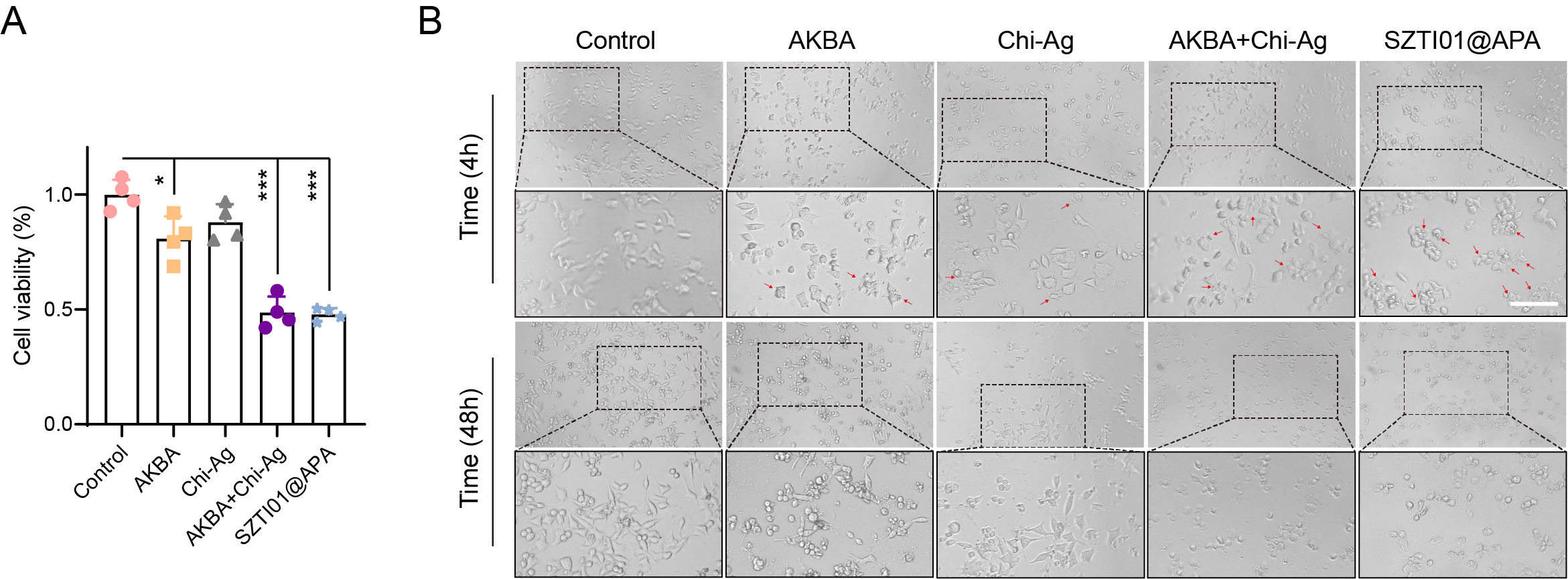


**Figure S5. *In vitro* antitumor activity of SZTI01@APA NPs. (A)** MTT assay of 22RV1 cells treated with PBS, AKBA, Chi-Ag NPs, AKBA+Chi-Ag NPs, and SZTI01@APA NPs for 48 h. **(B)** Light microscopy images showing the morphology of 22RV1 cells after 4 h and 48 h of intervention with PBS, AKBA, Chi-Ag NPs, AKBA+Chi-Ag NPs, and SZTI01@APA NPs. Scale bar: 100 μm.


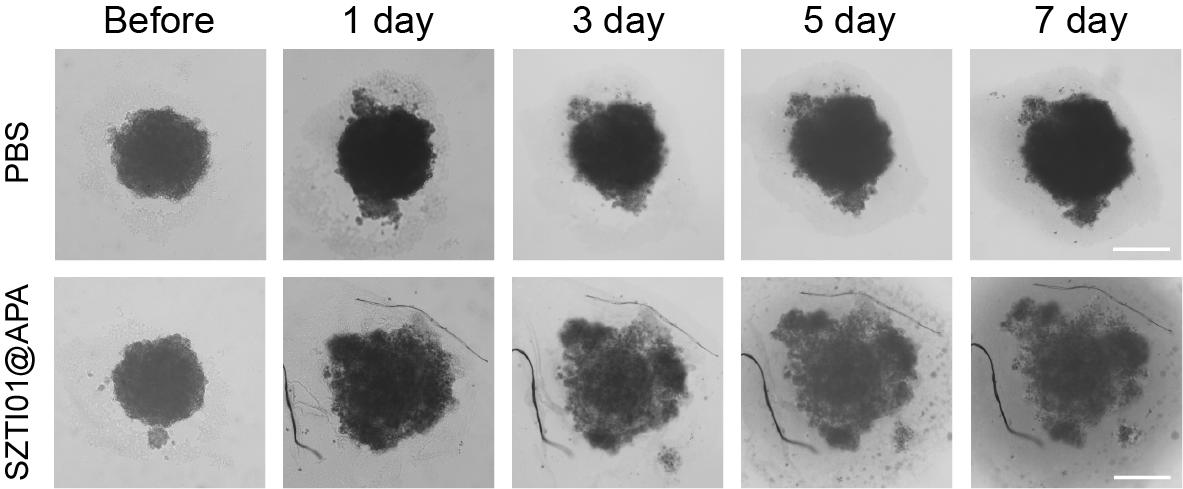


**Fig. S6.** Inhibition of multicellular spheroids growth was evaluated by treating with different treatments. Scale bar: 100 μm.


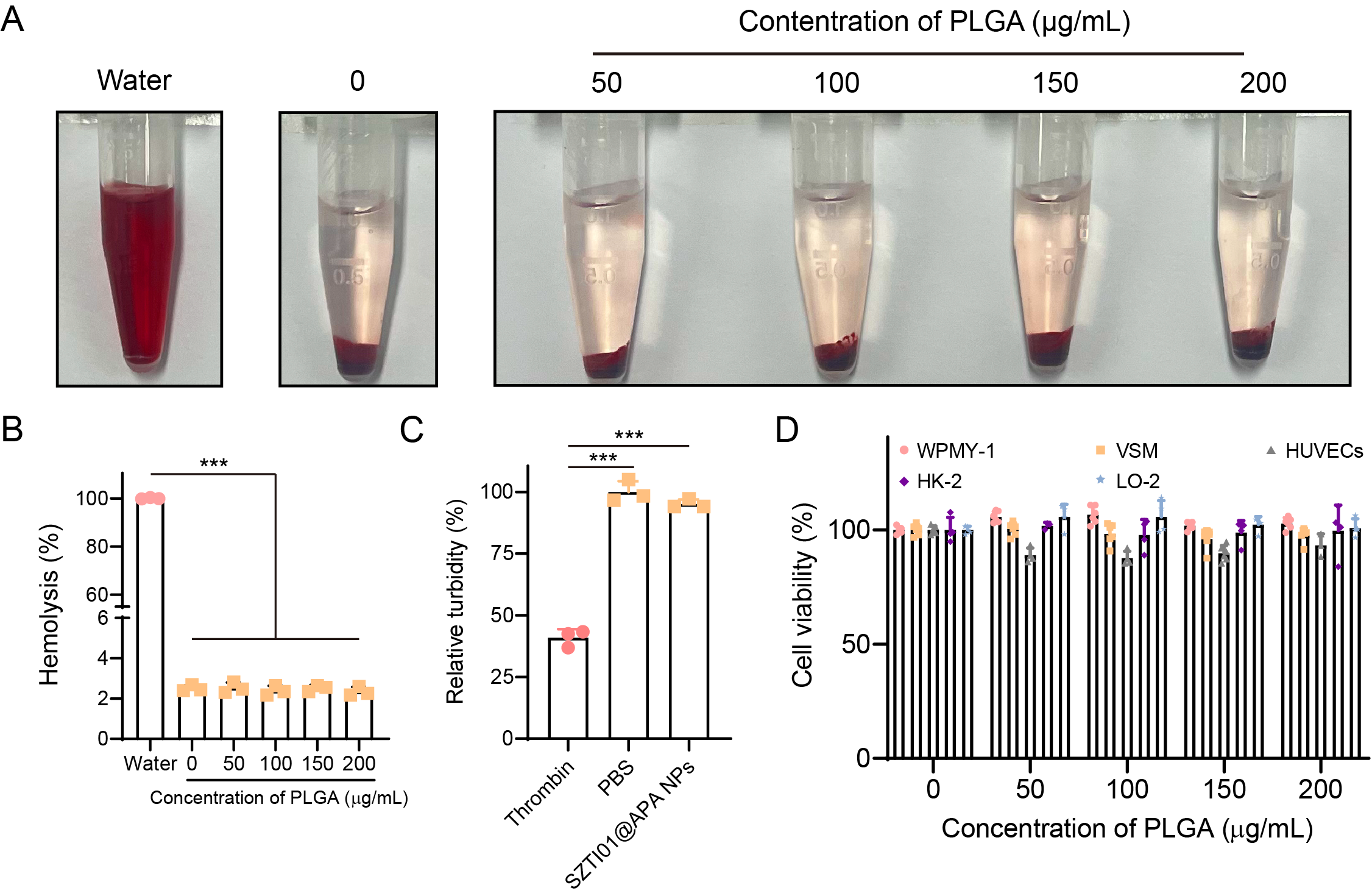


**Figure S7. Biocompatibility of SZTI01@APA NPs. (A-B)** Images and quantitative analysis of hemolysis after incubating red blood cells with various concentrations of SZTI01@APA NPs at 37°C for 4 h. **(C)** Coagulation assay displaying changes in platelet-rich plasma after treatment with thrombin and different concentrations of SZTI01@APA NPs. **(D)** Cell viability of different concentrations of SZTI01@APA NPs treated WPMY-1, VSM, HUVECs, HK2, and LO-2 cells for 24 h.


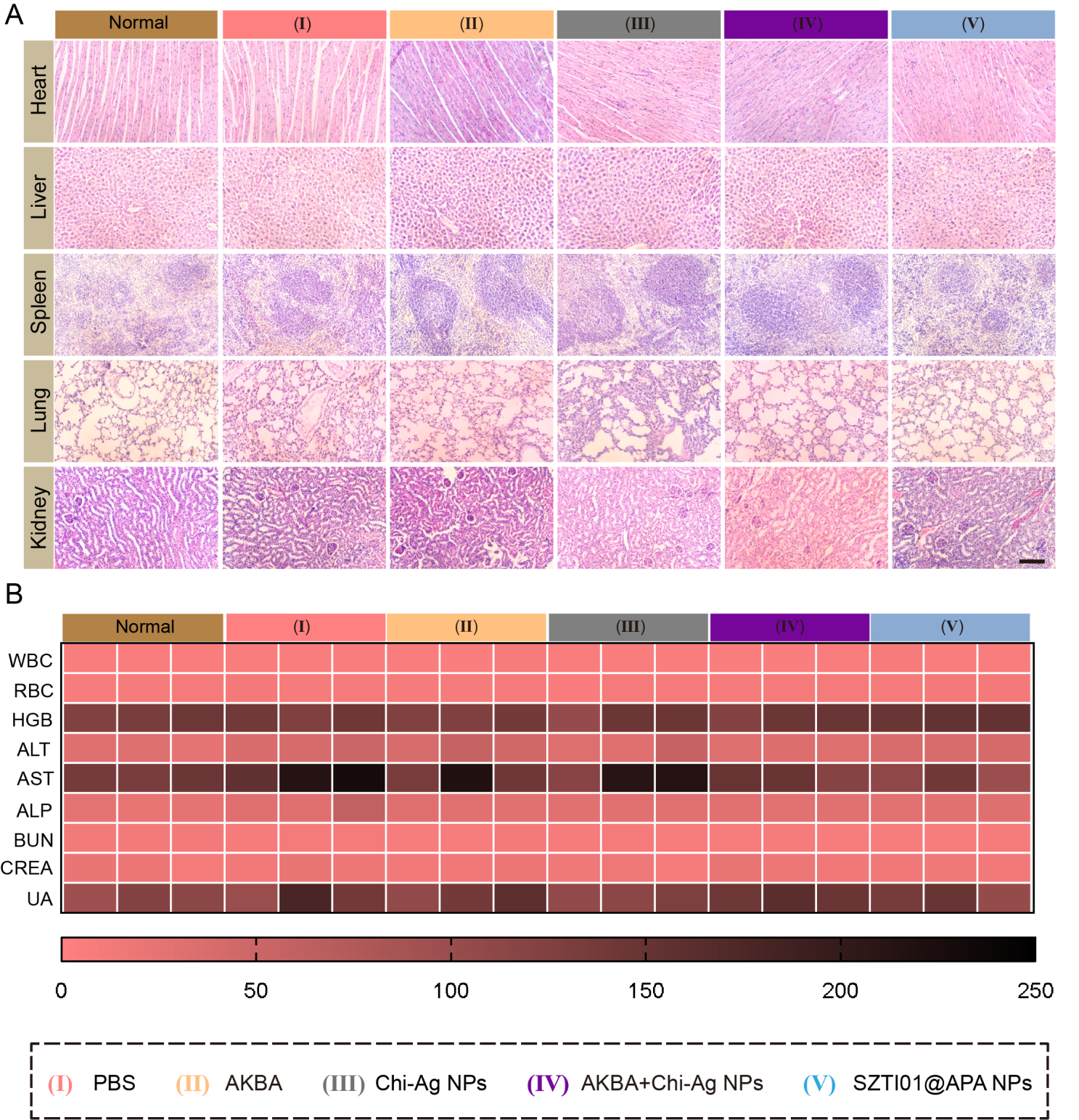


**Figure S8. Evaluation of in *vivo* toxicity of SZTI01@APA NPs. (A)** H&E staining images of the main organs (heart, liver, spleen, lung, and kidney) of nude mice in different treatment groups. Scale bar: 100 μm. **(B)** Blood routine analysis and renal function tests in nude mice from different treatment groups.


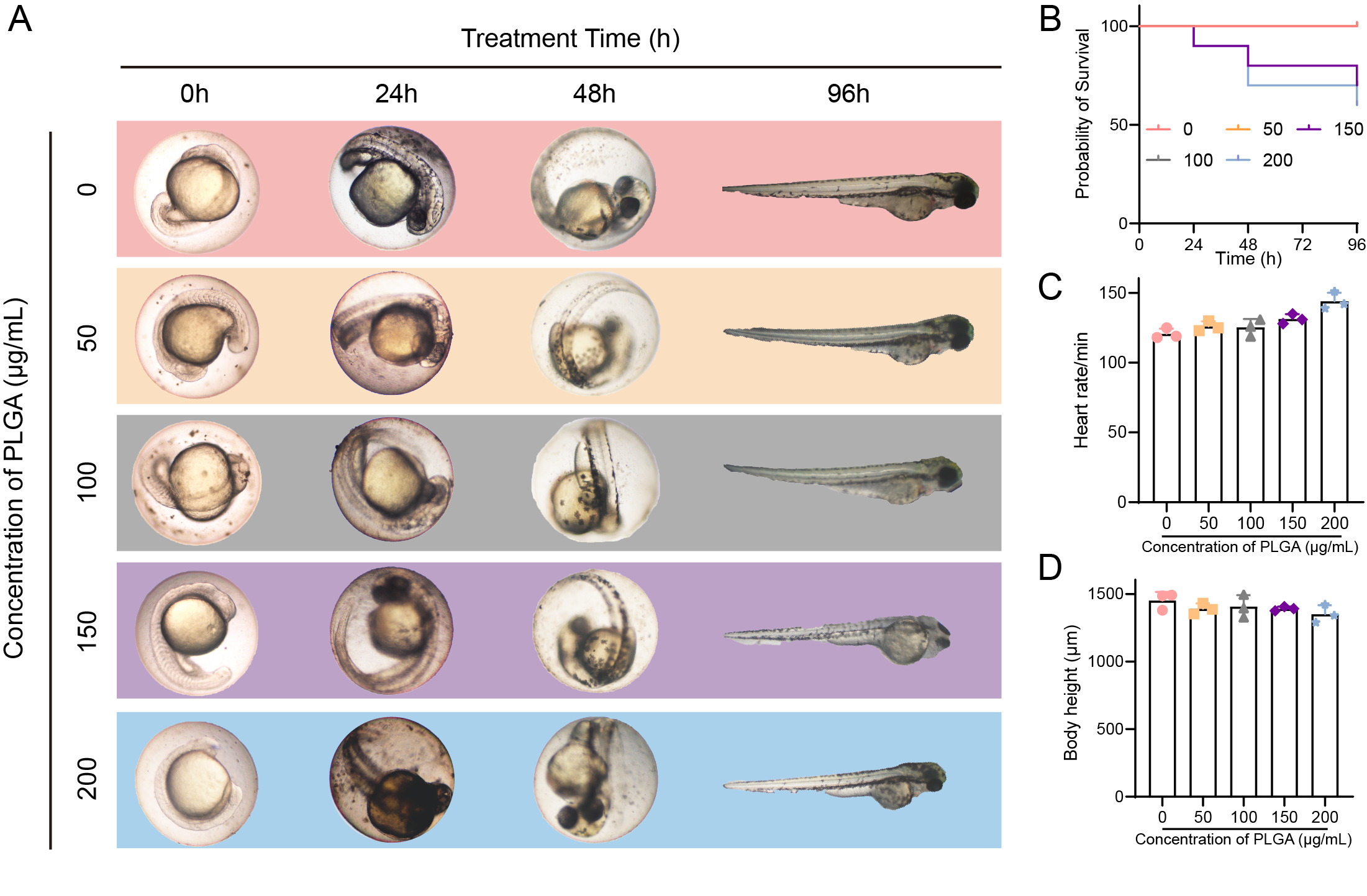


**Figure S9. Toxicity evaluation of SZTI01@APA NPs in zebrafish. (A)** Images of zebrafish embryos at different time points after intervention with different concentrations of SZTI01@APA NPs. **(B)** Survival rate of zebrafish embryos during a 96-hour incubation period. **(C)** Heart rate changes after co-incubation of zebrafish embryos with different concentrations of SZTI01@APA NPs for 72 h. **(D)** Effects of different concentrations of SZTI01@APA NPs on zebrafish body length.
